# An RNAi screen to identify proteins required for cohesion rejuvenation during meiotic prophase in *Drosophila* oocytes

**DOI:** 10.1093/g3journal/jkae123

**Published:** 2024-06-08

**Authors:** Muhammad A Haseeb, Alana C Bernys, Erin E Dickert, Sharon E Bickel

**Affiliations:** Department of Biological Sciences, Dartmouth College, Hanover, NH, USA 03755; Department of Biological Sciences, Dartmouth College, Hanover, NH, USA 03755; Department of Molecular Biology, Princeton University, Princeton, NJ, USA 08544; Department of Biological Sciences, Dartmouth College, Hanover, NH, USA 03755; Department of Pharmacology and Cancer Biology, Duke University, Durham, NC, USA 27710; Department of Biological Sciences, Dartmouth College, Hanover, NH, USA 03755

**Keywords:** *Drosophila*, meiosis, oocyte, sister chromatid cohesion, Brahma, Pumilio, Nipped-B

## Abstract

Accurate chromosome segregation during meiosis requires the maintenance of sister chromatid cohesion, initially established during premeiotic S phase. In human oocytes, DNA replication and cohesion establishment occur decades before chromosome segregation and deterioration of meiotic cohesion is one factor that leads to increased segregation errors as women age. Our previous work led us to propose that a cohesion rejuvenation program operates to establish new cohesive linkages during meiotic prophase in *Drosophila* oocytes and depends on the cohesin loader Nipped-B and the cohesion establishment factor Eco. In support of this model, we recently demonstrated that chromosome-associated cohesin turns over extensively during meiotic prophase and failure to load cohesin onto chromosomes after premeiotic S phase results in arm cohesion defects in *Drosophila* oocytes. To identify proteins required for prophase cohesion rejuvenation but not S phase establishment, we conducted a Gal4-UAS inducible RNAi screen that utilized two distinct germline drivers. Using this strategy, we identified 29 gene products for which hairpin expression during meiotic prophase, but not premeiotic S phase, significantly increased segregation errors. Prophase knockdown of Brahma or Pumilio, two positives with functional links to the cohesin loader, caused a significant elevation in the missegregation of recombinant homologs, a phenotype consistent with premature loss of arm cohesion. Moreover, fluorescence in situ hybridization confirmed that Brahma, Pumilio, and Nipped-B are required during meiotic prophase for the maintenance of arm cohesion. Our data support the model that Brahma and Pumilio regulate Nipped-B-dependent cohesin loading during rejuvenation. Future analyses will better define the mechanism(s) that govern meiotic cohesion rejuvenation and whether additional prophase-specific positives function in this process.

## Introduction

In both mitotic and meiotic cells, accurate chromosome segregation requires that sister chromatids remain physically associated from the time of their synthesis (S phase) until they segregate to opposite poles (McNicoll *et al.* 2013; Marston 2014; Morales and Losada 2018; Ishiguro 2019). In addition, during meiosis, cohesion between the arms of sister chromatids also provides an evolutionarily conserved mechanism to keep recombinant homologs associated until anaphase I (Buonomo *et al.* 2000; Bickel *et al.* 2002; Hodges *et al.* 2005). Within the cohesin complex, which mediates sister chromatid cohesion, the association of an α-kleisin subunit with the Smc1/Smc3 heterodimer results in the formation of a ring. Opening of the ring and topological entrapment of DNA by cohesin requires the cohesin loader (Scc2/Scc4 in yeast, NIPBL/MAU2 in mammals) (Alonso-Gil and Losada 2023). During DNA replication, the formation of stable cohesive linkages depends on the acetyltransferase (Eco1 in yeast, ESCO1/2 in mammals), which keeps the ring stably closed by acetylating two conserved lysines within the Smc3 head (Peters and Nishiyama 2012; Rankin and Dawson 2016).

In metazoans, one challenge oocytes face is that the formation of cohesive linkages during premeiotic S phase can occur days to decades prior to chromosome segregation, depending on the organism. Therefore, proper chromosome segregation in oocytes demands that a sufficient number of the original cohesive linkages remain intact or be replaced during the long prophase I arrest. Loss of cohesion in aging oocytes has been observed in multiple organisms (Chiang *et al.* 2012; Greaney *et al.* 2018; Wartosch *et al.* 2021; Charalambous *et al.* 2023), and cohesin turnover on meiotic chromosomes has not been detected in mouse oocytes (Revenkova *et al.* 2010; Tachibana-Konwalski *et al.* 2010; Burkhardt *et al.* 2016). These observations have led to the model that gradual deterioration of the original cohesive linkages in human oocytes contributes to increased segregation errors in the oocytes of older women, a phenomenon known as the maternal age effect.

*Drosophila* provides a powerful genetic system to dissect the mechanisms that influence cohesion maintenance in oocytes. Because the female germline matα-Gal4-VP16 driver is not expressed until after the completion of premeiotic S phase (Weng *et al.* 2014), one can use this driver to ask whether knockdown (KD) of a gene product exclusively during meiotic prophase disrupts the *maintenance* of cohesion in *Drosophila* oocytes. Using this strategy, we previously demonstrated that the knockdown of individual cohesin subunits, the *Drosophila* cohesin loader Nipped-B or the cohesion establishment factor Eco *after* premeiotic S phase results in phenotypes consistent with premature loss of cohesion (Weng *et al.* 2014). Based on these findings, we proposed that a cohesion rejuvenation program operates in *Drosophila* oocytes during meiotic prophase to establish new cohesive linkages that are required to maintain cohesion (Weng *et al.* 2014). In support of this hypothesis, we have recently reported that chromatin-associated cohesin turns over extensively during meiotic prophase in *Drosophila* oocytes (Haseeb *et al.* 2024). Moreover, failure to load cohesin onto oocyte chromosomes during meiotic prophase leads to premature loss of arm cohesion (Haseeb *et al.* 2024). These data provide evidence that de novo formation of cohesive linkages occurs after S phase in *Drosophila* oocytes and is required to maintain the association of sister chromatids and support accurate chromosome segregation during the meiotic divisions. Although it is possible that cohesion rejuvenation during meiotic prophase is specific to *Drosophila* oocytes, it is difficult to understand why this process would be necessary to maintain cohesion in fly oocytes during a 6-day timeframe, but not during the extended prophase arrest that lasts for months in mouse oocytes and years in human oocytes.

Understanding the mechanism(s) underlying cohesion rejuvenation requires the identification of the proteins involved, particularly those that may be unique to this process. Given that cohesion rejuvenation in the *Drosophila* oocyte occurs in the absence of global DNA replication, it likely differs mechanistically from the formation of stable cohesive linkages during S phase. In addition, we have previously shown that cohesion rejuvenation in prophase oocytes occurs in the absence of double-strand breaks (Weng *et al.* 2014) distinguishing it from the pathway that operates during G2 in mitotically dividing yeast cells subjected to DNA damage (Strom *et al.* 2004; Strom *et al*. 2007; Unal *et al.* 2007).

**Fig. 1.**
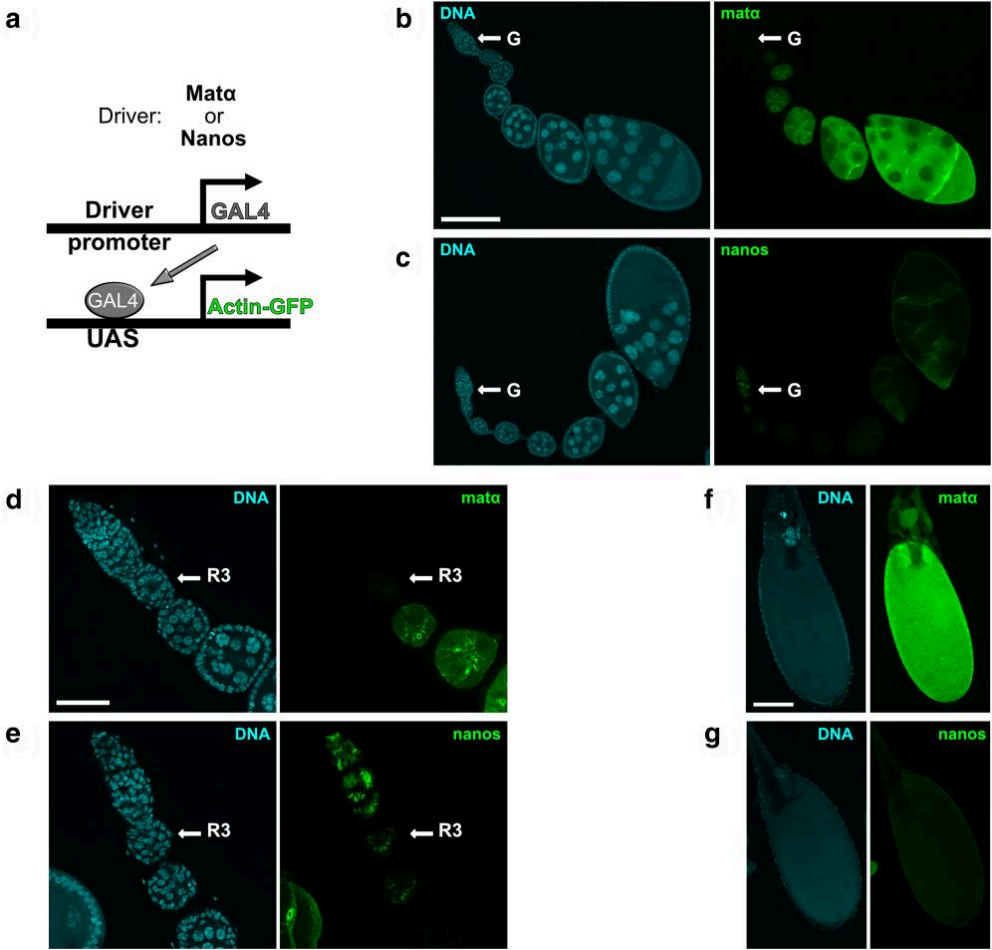
Comparison of matα-Gal4 and nanos-Gal4 expression patterns and relative strengths within *Drosophila* ovarioles. a) Schematic illustrates the Gal4-UAS method utilized to express GFP-tagged actin in *Drosophila* ovaries using two different germline-specific drivers, matα-Gal4-VP16 or nanos-Gal4-VP16. b–c) *Drosophila* ovarioles are shown for which UASp-actin-GFP expression (green) is induced by the matα or the nanos driver. DNA is shown in blue. The arrows indicate the germarium (G) at the anterior of each ovariole. Scale bar, 120 µm. To allow a comparison of relative driver strengths, images for both drivers were captured and processed identically in this and subsequent panels. All images are maximum intensity projections of confocal Z-series. d–e) Higher magnification images highlight the different expression patterns of the two drivers within the germarium. Region 3 (R3) of the germarium is labeled. Scale bar, 40 µm. Note that nanos-Gal4-induced expression is visible within several germline cysts of the germarium, including the stage at which meiotic DNA replication occurs. In contrast, the matα-Gal4 driver does not turn on until R3 or stage 2, approximately 2 days after premeiotic S phase. f–g) In mature oocytes (stages 13–14), the actin-GFP signal resulting from the matα driver is much stronger than that induced by the nanos driver. Scale bar, 120 µm.

**Fig. 2.**
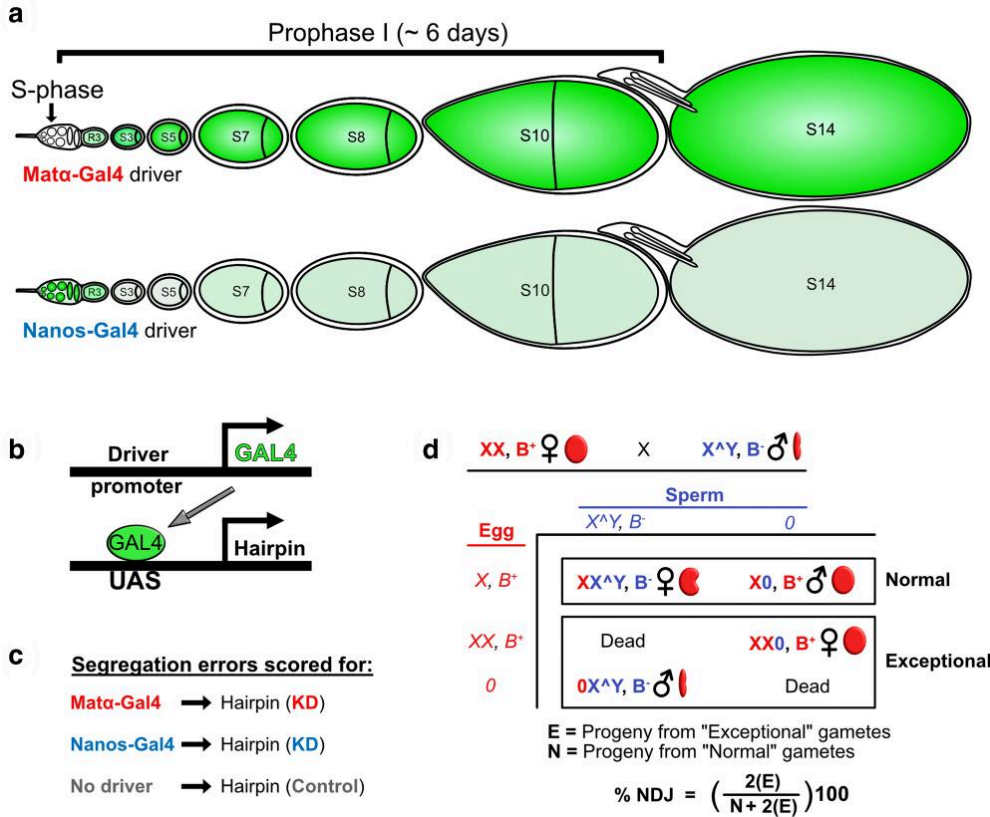
Experimental strategy to identify proteins required for cohesion rejuvenation during meiotic prophase. a) Cartoon representation of the *Drosophila* ovariole uses green shading to depict the expression patterns and relative strengths of the matα-Gal4 and nanos-Gal4 drivers. Upon completion of premeiotic S phase in the germarium, germline cysts enter meiotic prophase. Note that not all stages are present in a single ovariole at any given timepoint. b) UAS-Gal4 strategy utilizes the matα-Gal4 or nanos-Gal4 driver to express a specific hairpin in the female germline. c) Chromosome segregation errors in knockdown (KD) oocytes are quantified and compared to control oocytes containing the hairpin transgene but no driver. d) In the *X*-chromosome nondisjunction (NDJ) assay, KD or control females are crossed to males with an attached *X^Y* chromosome containing the dominant eye-shape marker, Bar. Based on their sex and eye shape, progeny arising from accurate *X*-chromosome segregation (normal, N) can be distinguished from those resulting from aberrant segregation (exceptional, E). Because only half of the exceptional gametes result in viable progeny, the value of E is doubled when calculating the total % NDJ.

With the goal of identifying proteins that are required for prophase rejuvenation but not S phase establishment, we designed a Gal4-UAS RNAi screen to quantify and compare chromosome segregation errors [nondisjunction (NDJ)] in control oocytes (hairpin but no driver), prophase KD oocytes (matα-Gal4-VP16 —> hairpin), and S phase KD oocytes (nanos-Gal4-VP16 —> hairpin) (see Figs. 1 and 2). We reasoned that a hairpin targeting a protein *specific* for cohesion rejuvenation should cause a significant increase in NDJ when expressed during meiotic prophase but not premeiotic S phase.

Here we report the results of our screen, including identification of 29 positives for which knockdown with the matα but not the nanos driver significantly increases meiotic chromosome segregation errors. Functional links to the cohesin loader have been reported for 2 prophase-specific positives, Brahma (Brm) and Pumilio (Pum). Knockdown of either protein during meiotic prophase caused a significant elevation in the frequency at which recombinant homologs missegregate, a phenotype consistent with premature loss of arm cohesion. Furthermore, direct analysis of the state of cohesion using fluorescence in situ hybridization (FISH) indicated that matα-induced KD of Brm or Pum causes a significant increase in oocytes with premature loss of arm cohesion. These results validate the general strategy of our screen and suggest that further analysis of additional positives will provide insight into the mechanism(s) underlying cohesion rejuvenation during meiotic prophase.

## Materials and methods

### Fly stocks and crosses

All fly stocks and crosses were maintained on standard cornmeal-molasses food at 25°C in a humidified incubator. Supplementary Table 1 provides genotypes and information for general stocks utilized in the screen and specific short hairpin stocks used for follow-up experiments. Supplementary Table 2 provides stock information for each of the hairpin stocks tested for NDJ in the primary screen.

### *X*-chromosome NDJ assay

Supplementary Fig. 1 shows representative crosses performed in parallel for each hairpin tested. Males carrying a UAS-hairpin transgene were crossed to *y w; +; mtrm*^KG^*matα-Gal4-VP16/TM3* (W-110), *nanos-Gal4-VP16; +; mtrm*^KG^*/TM3* (T-764), or *y w; +; mtrm*^KG^*/TM3* virgins (W-109). For the nondisjunction (NDJ) assay, nonbalancer virgin female progeny from the above crosses were mated to *X^Y, v f B* (C-200) males. For each of the three genotypes tested for each hairpin (Fig. 2c, Supplementary Fig. 1), 10 vials of the NDJ cross were started (8 virgins × 4 males), and oocyte segregation errors were measured by scoring the progeny through day 18 (Fig. 2d). *P*-values were calculated using the method described previously (Zeng *et al.* 2010).

### *X*-chromosome recombinational history and crossover frequency assays

To determine whether matα-induced KD of Brm or Pum increased missegregation of recombinant homologs, we utilized a genetic “recombinational history” assay that allows us to deduce whether an individual Diplo-*X* female arising from an NDJ event carries two homologs or two sister chromatids and whether one or both *X* chromosomes underwent a crossover before missegregation (Subramanian and Bickel 2008; Weng *et al.* 2014; Perkins *et al.* 2016). We created Brm and Pum hairpin stocks (I-563 and I-576, Supplementary Table 1) that contained an *X* chromosome marked with *y.* Virgins from these stocks were crossed to *y sc cv v f car/B^S^Y; + ; mtrm*^KG^*/TM3* (M-835) and *y sc cv v f car/B^S^Y; + ; matα mtrm*^KG^*/TM3* (M-834) males. The resulting *y/y sc cv v f car*; *mtrm*^KG^*/TRiP hairpin* (**control**) and *y/y sc cv v f car*; *matα mtrm*^KG^*/TRiP hairpin* (**KD**) virgins were crossed to *X^Y, v f B* males (C-200) and NDJ scored daily from day 10 through day 18. Diplo-*X* progeny were collected each day, phenotyped for *sc*, *cv*, *f*, and *car*, and each female mated to two *y w* males (A-062). By scoring her male progeny for *sc*, *cv*, *f*, and *car* and considering the phenotype of the Diplo-*X* female, we were able to deduce the genotype of the two *X* chromosomes she inherited, whether they were sisters or homologs (based on *car*) and whether either were recombinant (Supplementary Fig. 2). The frequency at which recombinant chromosomes missegregated was calculated by dividing the number of Diplo-*X* progeny that inherited at least one recombinant chromosome by the total number of progeny in the NDJ test and multiplying by 1,000 to facilitate comparisons. *P*-values were calculated using a two-tailed Fisher's exact test (GraphPad).

One limitation of this assay is that it underestimates the number of recombinant bivalents that underwent missegregation because only two of the four chromatids can be genotyped. In addition, double crossovers in the large interval between *cv* and *f* will be invisible to us. Finally, although the proximity of *car* to pericentric heterochromatin (3.5cM) makes crossovers unlikely, a small number may still occur.

To determine whether knockdown of Brm or Pum altered the frequency and/or distribution of *X* chromosome crossovers, we crossed *y/y sc cv v f car*; *mtrm*^KG^*/TRiP hairpin* (**control**) and *y/y sc cv v f car*; *matα mtrm*^KG^*/TRiP hairpin* (**KD**) virgins to *y w* males (A-062). Male progeny were scored for *sc*, *cv*, *f*, and *car*, and map distance was calculated for each interval. A two-tailed Fisher's exact test (GraphPad) was used to calculate significance.

### Whole mount ovary preparation for GFP-tagged actin reporter expression

To characterize the expression patterns and relative strengths of the two germline Gal4 drivers used in this study, we analyzed ovaries from flies that expressed GFP-tagged actin under the control of the matα or nanos-Gal4 driver. *y w; +; mtrm*^KG^*matα-Gal4-VP16/TM3* (W-110) or *nanos-Gal4-VP16; +; mtrm*^KG^*/TM3 (*T-764) males were mated to *y w; P{UASp-Act5C.T:GFP}2; +* (A-201) virgins. Nonbalancer young female progeny were held in food vials with males and dry yeast for 2 days before dissection in a shallow dish containing 1× PBS. The anterior region of each ovary was gentlysplayed open, and the ovaries were fixed for 5 min at room temperature in 1X PBS containing 4% formaldehyde (Ted Pella, 18505). After 3 rinses in 1× PBS, the ovaries were incubated in 1X PBS containing 2.0 µg/ml Hoechst 33342 (Molecular Probes H3570) with gentle shaking for 30 min. Following 3 rinses and a 15-min wash in 1× PBS, individual ovarioles were separated using tungsten needles and transferred to poly-L-lysine coated 18 mm #1.5 coverslips. For mounting, 25 µl of SlowFade Diamond Antifade (Molecular Probes S36967) was used and the edges of the coverslips were sealed with nail polish before imaging.

### FISH

We utilized FISH to quantify cohesion defects in KD and control oocytes containing Brm, Pum, or Nipped-B hairpin transgenes. To generate each pair of samples (KD and control), *y sc v; +; P{TRiP Brm^V20^}attP2* (H-209), *y sc v sev; +; P{TRiP Pum^V20^}attP2* (H-211), or *y sc v; P{TRiP Nipped-B*^V22^*}attP40; +* (H-063) virgins were crossed to *w; +; P{matα-GAL4-VP16}V37* (T-273) or *y w; +; +* (A-062) males. Note that oocytes used for all FISH experiments were wild-type for *mtrm*. Young female progeny were held in food vials with males and yeast for 3 days before ovaries were dissected. After fixation, stage 13–14 oocytes were processed as previously described (Perkins *et al.* 2016; Perkins and Bickel 2017). Fixation, predenaturation, hybridization, washes, and mounting were performed as reported previously (Haseeb *et al.* 2024) except that ovaries were fixed for 6 min instead of 4 min. To monitor arm cohesion, we utilized an Alexa 647-labeled Oligopaint probe (OPP122 from Joyce Lab, University of Pennsylvania) comprised of a mixture of 80-base oligonucleotides that hybridize across a 100-kb distal region on the *X* chromosome. Cohesion within the pericentric heterochromatin was analyzed using a Cy3-conjugated probe (5′-Cy3-AGGGATCGTTAGCACTCGTAAT; Integrated DNA Technologies) that targets an 11Mb region of satellite DNA on the *X* chromosome (Dernburg 2000). Arm and pericentric probes were used at final concentrations of 0.50 pmol/µl and 1 ng/µl, respectively. Following image acquisition, cohesion defects were scored, blind to genotype, as detailed previously (Haseeb *et al.* 2024). A two-tailed Fisher's exact test (GraphPad) was used to determine the statistical significance of differences between KD and control.

### Image acquisition and analysis

All images were acquired using an Andor spinning disk confocal on a Nikon Eclipse Ti inverted microscope equipped with an ASI MS-2000 motorized piezo stage, a 50-µm pinhole disk, and a Zyla 4.2-mega pixel sCMOS camera. Nikon Elements software (version 5.11.02 Build 1369) and up to 4 lasers (405, 488, 561, and 637 nm) were used for image acquisition. For Fig. 1, d and e, we utilized a Nikon CFI 40× Plan Fluor oil objective (NA 1.3) to acquire a specified region of interest (ROI). For Fig. 1, b, c, f, and g, full-frame images were captured using a Nikon CFI 20× Plan Apo objective (NA 0.75). All FISH images were collected using a Nikon CFI 100× oil Plan Apo DIC objective (NA 1.45). Frame averaging (4×) was employed for all image acquisition. For the Z-series, a step size of 1 µm, covering an 8-µm range, was used for Fig. 1 and Supplementary Fig. 3, and a 0.1-µm step size over a 4-µm range was utilized for FISH imaging. Starting with the longest wavelength, an entire Z-stack was acquired with one laser before proceeding to the next channel. For Fig. 1 and Supplementary Fig. 3, images were acquired and processed identically for both matα-Gal4 and nanos-Gal4 driven UASp-actin-GFP, including the number of optical sections included in the projections in Fig. 1.

### Quantification of matα and nanos driver strengths at different stages

Actin-GFP signal intensity (488 nm) was quantified at different stages in matα —> UASp-Actin-GFP and nanos —> UASp-Actin-GFP fixed ovarioles using Volocity Quantification (v6.5.0). For full-frame ovariole images (20× objective), a free-hand tool was utilized to select a specific ROI while viewing a maximum intensity projection in the DAPI channel. For the germarium, the ROI included all cells except the region 3 cyst. For stages 3–4 and 7–8, the ROI included only germline cells and excluded follicle cells. Egg chambers were staged using morphological criteria and size (King 1970; Mahowald and Kambysellis 1980; Spradling 1993). For stages 13–14, the ROI included the entire oocyte but not the dorsal appendages. For each ovariole image, an ROI was drawn in an area lacking tissue and used to determine the background signal intensity. The average intensity for all voxels within each ROI was calculated, and a box and whisker graph was generated (Microsoft Excel) to present the data for “background” as well as each of the stages quantified (Supplementary Fig. 3). *P*-values were calculated using an unpaired *t* test in Microsoft Excel, with *P* < 0.05 considered significant.

## Results and discussion

### Screen rationale and design

To screen for proteins required for cohesion maintenance during meiotic prophase, we utilized two different germline-specific Gal4-VP16 drivers to induce the expression of RNAi hairpins at different times during *Drosophila* oogenesis. Figure 1 and Supplementary Fig. 3 provide a comparison of the relative strengths and expression patterns of the matα-Gal4-VP16 and nanos-Gal4-VP16 drivers (hereafter referred to as matα and nanos drivers). Expression of the matα driver is first detectable in germarial region 3 (Weng et al. 2014) or stage 2 of the ovariole (Fig. 1), approximately 2 days after completion of premeiotic S phase. Therefore, knockdown using this driver is restricted to meiotic prophase. In contrast, the nanos driver is expressed in multiple mitotic and meiotic cysts within the germarium, including the cells undergoing premeiotic S phase (Fig. 1). Although the nanos-Gal4-induced expression is also detectable during mid to late prophase, it is significantly weaker than that for the matα driver (Supplementary Fig. 3).

We utilized *mtrm^KG08051/+^* heterozygotes as a sensitized genetic background for our screen. Matrimony protein is required for accurate segregation of achiasmate bivalents in *Drosophila* oocytes. This achiasmate segregation system, which relies on pericentric heterochromatin mediated association of homologs (Hawley *et al.* 1992; Karpen *et al.* 1996), also ensures proper segregation of crossover homologs that lose their physical connection due to premature loss of arm cohesion (Supplementary Fig. 4). Therefore, the hairpin expression that causes premature loss of meiotic cohesion may not significantly elevate NDJ if oocytes are wild-type for *mtrm.* Because the achiasmate pathway is disabled in *mtrm^KG/+^* oocytes (Harris *et al.* 2003), our ability to identify gene products required for cohesion maintenance is enhanced in this genotype. In addition to its role in achiasmate segregation, Mtrm also promotes maintenance of sister chromatid cohesion in late prophase by binding and inhibiting Polo kinase (Xiang *et al.* 2007; Bonner *et al.* 2020; Haseeb *et al.* 2024). Although we have observed (using FISH) that arm cohesion defects are higher in *mtrm^KG/+^* oocytes than *mtrm^+^* oocytes, weak expression of a hairpin that targets the cohesin subunit Smc3 significantly increases cohesion defects in *mtrm^KG/+^* oocytes (Haseeb *et al.* 2024). Therefore, weak cohesion defects in KD oocytes will likely be amplified in *mtrm^KG/+^* oocytes, increasing the likelihood of elevated NDJ (Supplementary Fig. 4).

To generate a list of potential gene products to knock down in our screen, we searched FlyBase for genes expressed in the female germline (oocyte and/or nurse cells). For each candidate on this list, we determined whether a Valium 20 or 22 insertion was available from the TRiP (Transgenic RNAi Project) collection (Ni *et al.* 2011). These vectors provide robust expression of a short hairpin and effective knockdown in the germline. We favored UAS hairpins inserted at the attP2 site (chromosome *3*) but also tested a few attP40 insertions (chromosome *2*). For each candidate tested, we performed crosses (Supplementary Fig. 1) to generate control (no KD), matα-driven KD, and nanos-driven KD oocytes and performed our NDJ assay in parallel for all three genotypes (Fig. 2).

### Hairpins for which nanos-Gal4-induced knockdown causes a significant increase in meiotic segregation errors

**Fig. 3.**
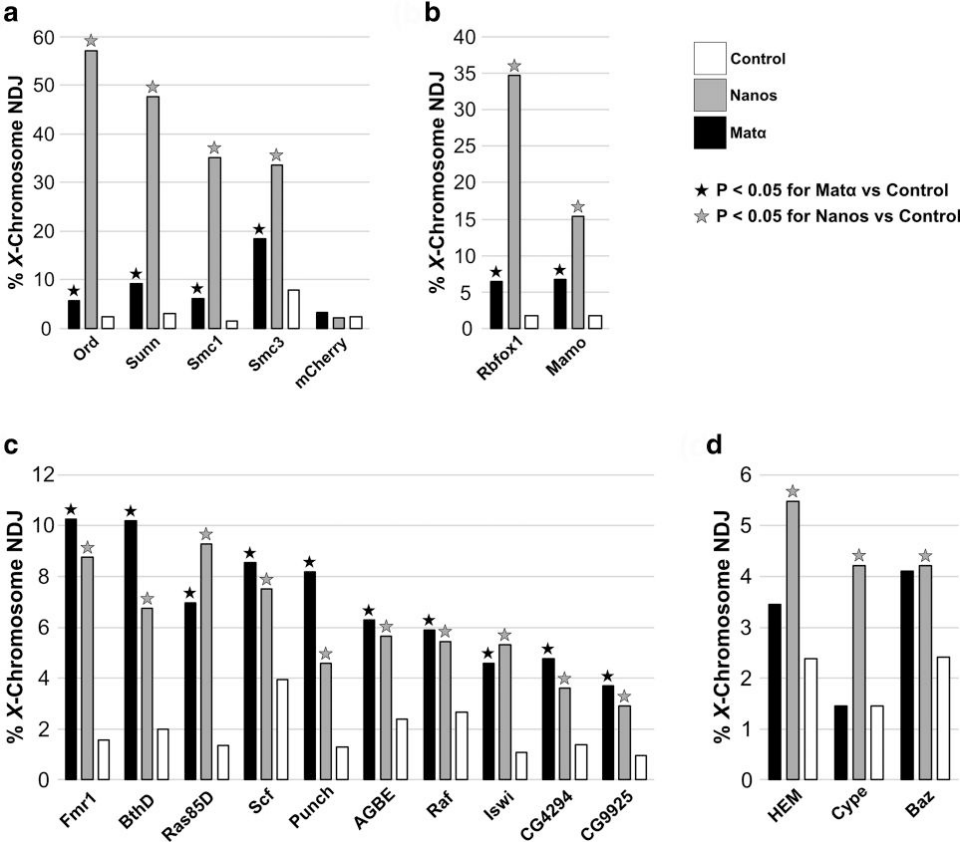
Gene products for which nanos-induced knockdown (KD) causes a significant elevation in NDJ. Histogram compares *X*-chromosome NDJ in no driver —> control (white), nanos-Gal4 —> KD (gray), and matα-Gal4 —> KD (black) oocytes. A star indicates a significant difference (*P* < 0.05) between the NDJ in control oocytes and those with the indicated driver (nanos, gray star; matα, black star). a) NDJ increases significantly when either the nanos or matα driver is used to knock down *Drosophila* proteins known to be required for meiotic cohesion. However, NDJ is significantly higher with S phase KD than prophase KD (Supplementary Table 3). The expression of an mCherry hairpin serves as a negative control. b) Knockdown of Rbfox1 or Mamo using either driver causes a significant increase in NDJ but segregation errors are significantly greater for nanos-induced KD than for matα-induced KD (Supplementary Table 3). c) Knockdown using either driver elicits a significant increase in NDJ that is comparable for the two drivers (Supplementary Table 4). d) Gene products for which knockdown using the nanos driver but not the matα driver significantly elevates NDJ (Supplementary Table 5).

Given that the cohesin complex is required for cohesion establishment during S phase, we utilized hairpins targeting the cohesin subunits Smc1 and Smc3 to first validate our approach. Figure 3a compares NDJ in oocytes in which one of these cohesin subunits was knocked down using either the nanos or matα driver. Relative to control oocytes, nanos-induced KD of either cohesin subunit resulted in a robust, statistically significant increase in NDJ, consistent with their essential role during S phase cohesion establishment. In addition, as we have reported previously (Weng *et al.* 2014), matα-induced KD of Smc1 or Smc3 also caused a significant elevation in meiotic NDJ compared to control, consistent with their requirement for cohesion rejuvenation during meiotic prophase. Notably, for both Smc1 and Smc3, NDJ was significantly higher for nanos KD than matα KD (see Supplementary Table 3 for *P*-values). We consider the above phenotypes to be a useful reference indicative of gene products that are required for both S phase establishment and prophase rejuvenation in *Drosophila* oocytes.

As a negative control, we expressed a Valium 20 hairpin targeting mCherry in flies that lack a mCherry-encoding transgene (Fig. 3a). Compared to the no driver control, meiotic NDJ was not significantly elevated with either the nanos or the matα drivers in *mtrm^KG/+^* heterozygotes containing the mCherry hairpin (Fig. 3a, Supplementary Table 3). These results strengthen our confidence that candidates uncovered in our screen are not false positives.

As part of our validation strategy, we also investigated two other proteins required for meiotic cohesion in *Drosophila* for which Valium 20–22 hairpin stocks are available. Null mutations in the *orientation disruptor* (*ord*) gene cause segregation defects consistent with complete loss of meiotic cohesion in both oocytes and spermatocytes (Bickel *et al.* 1997). Although the molecular function of Ord is not fully understood, Ord protein localizes to both the arms and centromeres of meiotic chromosomes in *Drosophila* oocytes (Khetani and Bickel 2007), is required for chiasma maintenance (Bickel *et al.* 2002) and for localization of Smc1 and Smc3 to oocyte centromeres (Webber *et al.* 2004). Mutations in *sisters unbound* (*sunn*) also disrupt meiotic cohesion in both oocytes and spermatocytes (Krishnan *et al.* 2014), and Sunn protein has been proposed to function within one of the two meiosis-specific cohesin complexes in *Drosophila* (Krishnan *et al.* 2014; Gyuricza *et al.* 2016). Like Smc1 or Smc3 KD oocytes, meiotic NDJ is significantly elevated when Ord or Sunn is knocked down using either the nanos or the matα driver (Fig. 3a). Moreover, chromosome segregation errors are significantly more prevalent following S phase KD than prophase KD (see Supplementary Table 3 for *P*-values). The data obtained with the nanos driver were not unexpected and indicate that like Smc1 and Smc3, both Ord and Sunn are essential for cohesion establishment during premeiotic S phase in *Drosophila* oocytes. In addition, our results using the matα driver suggest that, similar to Smc1 and Smc3, Ord and Sunn proteins are also required during meiotic prophase for cohesion rejuvenation.

Interestingly, our screen uncovered two gene products for which knockdown resulted in phenotypes similar to those of the cohesion proteins tested above (Fig. 3b, Supplementary Table 3). Compared to control oocytes, nanos and matα drivers both caused a significant increase in meiotic NDJ in Rbfox1 (RNA-binding Fox protein 1) and Mamo (maternal gene required for meiosis) KD oocytes. Although neither protein has been implicated in cohesion regulation, Mamo is required for normal chromatin structure in *Drosophila* oocytes (Mukai *et al.* 2007; Hira *et al.* 2013) and Rbfox1 regulates the translation of Pumilio, one of the prophase-specific positives we discuss below (Carreira-Rosario *et al.* 2016). Our results raise the possibility that these proteins play a role in cohesion establishment during premeiotic S phase as well as cohesion rejuvenation during meiotic prophase.

For 10 positives, NDJ was significantly elevated for both nanos and matα drivers (Fig. 3c); however, for all but Punch, segregation errors did not differ significantly between the two drivers (Supplementary Table 4). In addition, nanos-induced KD of these proteins resulted in NDJ that was considerably lower than that observed for Smc1 or Smc3 KD (approximately 4–10 fold lower). The positives presented in Fig. 3c have diverse functions and include two uncharacterized gene products (CG4294 and CG9925). We cannot rule out the possibility that the activity of these proteins during premeiotic S phase is necessary for accurate chromosome segregation. However, although nanos-driven UASp-actin-GFP expression during prophase is considerably weaker than that for the matα driver (Supplementary Fig. 3), signal is visible after exit from the germarium (Fig. 1e) and increases slightly in late prophase (Fig. 1c and Supplementary Fig. 3). Therefore, weak knockdown of these proteins during meiotic prophase (not S phase) may result in the increased NDJ observed with the nanos driver.

Our screen also uncovered three proteins for which knockdown with the nanos but not the matα driver caused a relatively small but significant increase in *X*-chromosome NDJ compared to no driver controls (Fig. 3d, Supplementary Table 5). Because the much stronger matα driver (Supplementary Fig. 3) did not significantly increase NDJ compared to control oocytes, the phenotype obtained with the nanos driver is consistent with an essential role during premeiotic S phase. However, because expression of the nanos driver is not restricted to premeiotic S phase, we cannot rule out an early prophase function or the possibility that knockdown in the mitotic cysts of the germarium is responsible for the increased meiotic NDJ we observe with the nanos driver.

### Prophase-specific positives

**Fig. 4.**
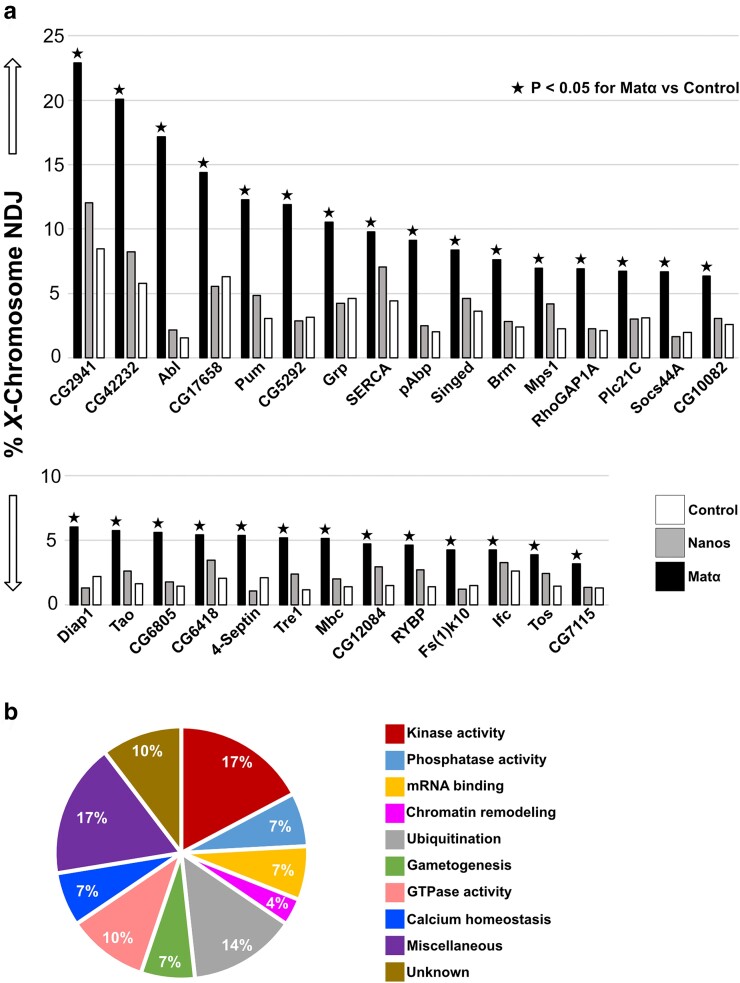
Proteins for which matα−induced but not nanos-induced knockdown causes meiotic NDJ to increase significantly. Histogram compares *X*-chromosome NDJ in no driver —> control (white), nanos-Gal4 —> KD (gray), and matα-Gal4 —> KD (black) oocytes. a) A significant increase in NDJ with the matα (black star) but not the nanos driver (no star) is consistent with a rejuvenation-specific role that is not required for cohesion establishment during oocyte DNA replication. b) Best-described cellular function for each of the prophase-specific positives is presented with a corresponding pie chart that indicates the relative percentage of positives in each category (also see Supplementary Table 8).

Of the 63 hairpin targets that we tested, knockdown of 29 proteins resulted in a significant increase in NDJ with the matα driver but not the nanos driver (Fig. 4a, Supplementary Table 6). This prophase-specific phenotype is what we would expect for proteins that are required for cohesion rejuvenation during meiotic prophase and not for cohesion establishment during premeiotic S phase. Although matα-induced KD uncovered several negatives (Supplementary Table 7), the high percentage of prophase-specific positives was unexpected (Fig. 4, Supplementary Table 6). However, because accurate chromosome segregation in oocytes depends on several pathways in addition to cohesion maintenance, it is unlikely that increased NDJ for all these hits arises because of premature loss of meiotic cohesion. As shown in Fig. 4b and Supplementary Table 8, the prophase-specific positives function in a diverse array of cellular pathways/mechanisms and include nine uncharacterized gene products. Most of these rejuvenation candidates exhibit less than 10% NDJ when knocked down using the matα driver, with only seven positives that exceed that value (Fig. 4a). However, this relatively low level of NDJ may still reflect a bona fide role in cohesion rejuvenation given that it is comparable to what we observe for matα-induced KD of three of the four cohesion proteins we tested (Ord, Sunn, and Smc1; Fig. 3a). Although four hairpins (Hang, eIF5, Dhc64C, Hip14) resulted in a significant NDJ increase for matα− but not nanos-induced KD, we did not graph these as prophase-specific positives in Fig. 4 because nanos —> KD oocytes were sterile or nearly sterile (Supplementary Table 6). Such a phenotype is consistent with an essential role during premeiotic S phase.

For a subset of the positives shown in Fig. 4, we tested a second hairpin to potentially rule out off-target effects. For Abl (Ableson kinase), Brm (Brahma), Mps1 (Monopolar spindle 1), and CG5292, NDJ arising with the second hairpin was consistent with our initial observations, significantly increased with the matα but not the nanos driver (Supplementary Table 6). For CG10081 and CG6805, neither the matα nor the nanos driver caused a significant increase in NDJ with the second hairpin (Supplementary Table 6). However, for these two uncharacterized genes, we cannot distinguish between an off-target effect with the first hairpin or insufficient KD with the second hairpin to elicit a significant increase in NDJ. Interestingly, the 2nd hairpin we utilized to knock down Pum (Pumilio) resulted in a significant NDJ elevation with the nanos driver as well as the matα driver, but NDJ was significantly lower for nanos-induced KD than for matα-induced KD (Supplementary Table 6). As discussed above for the positives in Fig. 3c, this phenotype could arise because of nanos driver activity in prophase and not reflect an essential role during premeiotic S phase.

### Brahma and Pumilio are required to maintain arm cohesion during meiotic prophase

Because several mechanisms govern accurate chromosome segregation, we set out to determine whether the NDJ we observed for a subset of prophase-specific positives occurs due to premature loss of cohesion. We selected Brahma (Brm) and Pumilio (Pum) for further analyses because each has published functional links with the cohesin loader (Gerber *et al.* 2006; Munoz *et al.* 2019; Munoz *et al.* 2020). Brm is the ATPase subunit of two chromatin remodeling complexes in *Drosophila* (Clapier and Cairns 2009). In yeast, the remodeling complex that contains the Brm ortholog facilitates cohesin loading to nucleosome-free regions by recruiting the cohesin loader Scc2 (Nipped-B in *Drosophila*) (Munoz *et al.* 2019; Munoz *et al.* 2020). Pum belongs to the evolutionarily conserved PUF family of sequence-specific RNA-binding proteins that control protein abundance by regulating mRNA stability and/or translation (Nishanth and Simon 2020). Interestingly, Pum has been shown to bind Nipped-B mRNA in *Drosophila* ovary extracts (Gerber *et al.* 2006).

**Fig. 5.**
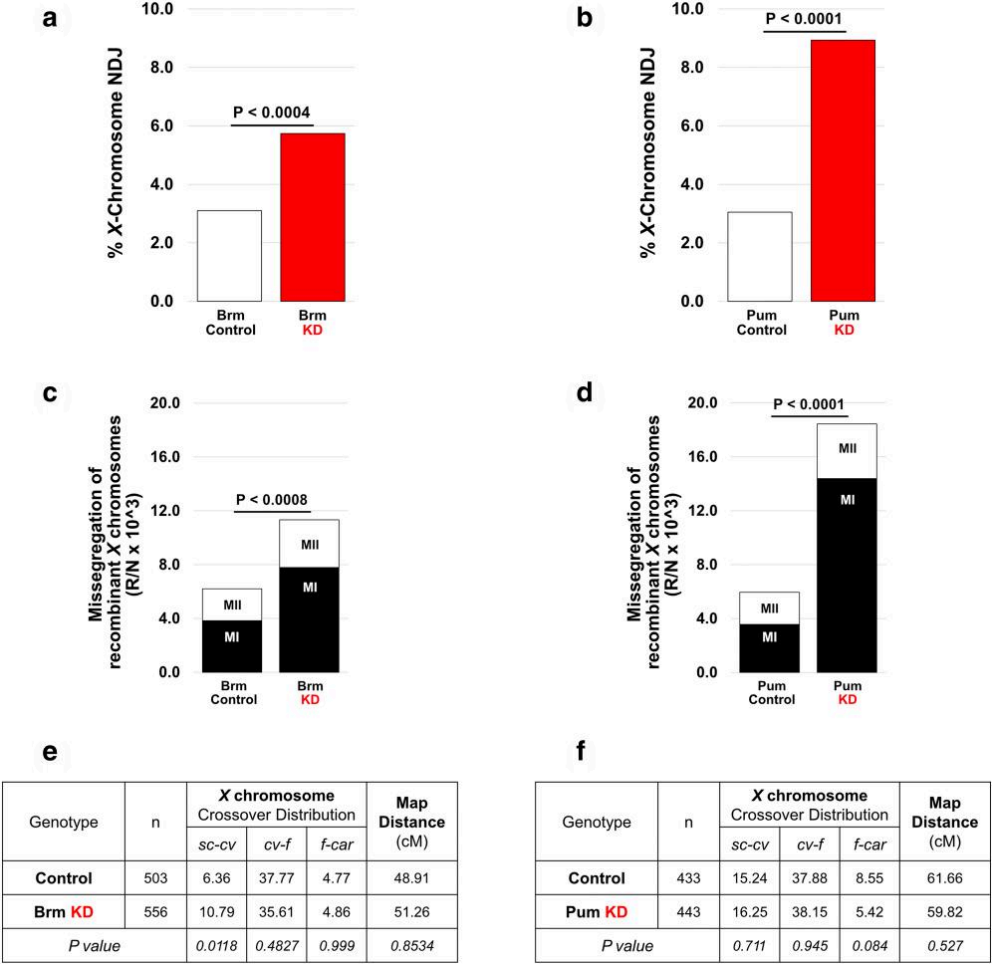
Knockdown (KD) of Brm or Pum increases missegregation of recombinant homologs without affecting crossover frequency. The matα driver and hairpins SH00130.N or SH02112.N were used to knock down Brm or Pum, respectively. a–b) *X*-chromosome NDJ tests for Brm KD and Pum KD (red bars) and their respective controls (white bars) yielded results consistent with those presented in Fig. 4. Diplo-*X* progeny from the NDJ tests were used for the subsequent *X*-chromosome recombinational history assay. c and d) The frequency at which Brm or Pum KD causes missegregation of recombinant *X* chromosomes is graphed. To calculate frequency, the number of Diplo-*X* females that received at least one recombinant chromosome (R) is divided by the total number of progeny (N) and multiplied by 1,000 to simplify the presentation. Inheritance of two homologs (MI segregation error) is depicted in black, and inheritance of two sisters (MII segregation error) is depicted in white. Matα-induced KD of either Brm or Pum caused a significant increase in the missegregation of recombinant homologs. *P*-values for MI errors are shown above the bars. Differences in the frequency of MII errors between KD and control were not significant for either Brm or Pum. e and f) Crossovers were scored within three intervals along the *X* chromosome. The map distance (cM) is presented for each interval as well as the total number of male progeny scored for each genotype (n). Matα-induced KD of Brm or Pum does not significantly alter the total map distance on the *X* chromosome (two-tailed Fisher's exact test).

Given that premature loss of arm cohesion leads to chiasma destabilization (Buonomo *et al.* 2000; Bickel *et al.* 2002; Hodges *et al.* 2005), we asked whether prophase KD of Brm or Pum increases missegregation of recombinant homologs (Supplementary Fig. 2). Using females that were heterozygous for recessive visible markers along the *X* chromosome, we repeated the NDJ assay and again observed a significant elevation in segregation errors in matα KD oocytes compared to their respective controls (Fig. 5, a and b). Following the NDJ assay, we performed an additional cross and used the *X* chromosome visible markers to genotype the male progeny for each Diplo-*X* female and determine whether either of the missegregating *X* chromosomes that she inherited were recombinant (Supplementary Fig. 2). The centromere-proximal marker *carnation* allowed us to determine whether the Diplo-*X* female inherited two homologs (*car^+/−^,* MI error) or two sisters (*car^+/+^* or *car^−/−^*, MII error). We found that prophase KD of either Brm or Pum significantly increased the frequency at which Diplo-*X* females inherited two homologs (MI errors), at least one of which was recombinant (Fig. 5, c and d). MII errors (sisters) arising from a recombinant bivalent were not significantly different between KD and control oocytes (Fig. 5, c and d). Furthermore, the total map distance of the *X* chromosome was not significantly altered in KD oocytes (Fig. 5, e and f), indicating that missegregation of recombinant homologs is not elevated in Brm and Pum KD oocytes due to increased crossovers in these genotypes. Together, these data support the hypothesis that Brm and Pum are required for accurate chromosome segregation in *Drosophila* oocytes because they promote cohesion rejuvenation during meiotic prophase.

**Fig. 6.**
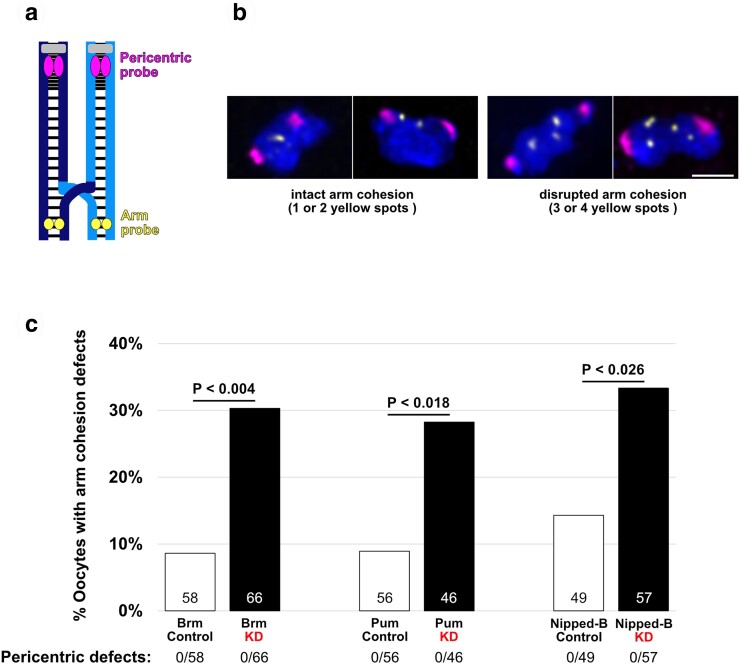
Prophase-specific positives Brm and Pum are required for cohesion maintenance during meiotic prophase. a) Cartoon depicts chiasmate *X-*chromosome bivalent with intact sister chromatid cohesion. Each homolog (dark blue and light blue) is composed of two sister chromatids with sister cohesion represented by the black lines and centromeres in gray. The magenta and yellow spots indicate the locations to which the FISH probes hybridize. b) Representative images illustrate intact or premature loss of arm cohesion in mature oocytes. Images are maximum intensity projections of deconvolved confocal Z-series. Scale bar, 2 µm. c) Quantification of arm cohesion defects in matα-Gal4 —> knockdown (KD) and control (no driver) oocytes. Number of oocytes scored for each genotype is shown within each bar. No defects in pericentric cohesion were observed in any of the six genotypes tested. A two-tailed Fisher's exact test was used to calculate *P*-values.

To directly assay whether sister chromatid cohesion is prematurely disrupted when Brm or Pum are knocked down during prophase, we performed FISH on mature *Drosophila* oocytes (stages 13–14). Using two different probes, we scored for cohesion defects within the pericentric heterochromatin as well as a distal region on the arm of the *X* chromosome (Fig. 6, a and b). All FISH experiments utilized oocytes that were wild-type for *matrimony*. When we knocked down Brm during meiotic prophase, the percentage of oocytes with arm cohesion defects increased significantly (Fig. 6c). Similarly, Pum KD caused a significant elevation in oocytes with premature loss of arm cohesion (Fig. 6c). We did not detect cohesion defects in pericentric heterochromatin for either of the KD or control genotypes tested. These data indicate that Brm and Pum are required during meiotic prophase in *Drosophila* oocytes to maintain arm cohesion between sister chromatids, potentially by influencing Nipped-B-dependent cohesin loading during rejuvenation.

Given our previous work implicating the cohesin loader in prophase rejuvenation (Weng *et al.* 2014) and its functional connection with Pum and the yeast ortholog of Brm (Gerber *et al.* 2006; Munoz *et al.* 2019; Munoz *et al.* 2020), we performed FISH to directly assess the state of sister chromatid cohesion in Nipped-B KD and control oocytes. Compared to the control, the percentage of Nipped-B KD oocytes exhibiting arm cohesion defects was significantly higher (Fig. 6c). Therefore, our FISH data confirm that Nipped-B is required during meiotic prophase for rejuvenation of arm cohesion in *Drosophila* oocytes. Similar to our findings for Brm and Pum, we did not observe defects in pericentric cohesion in Nipped-B KD or control oocytes. This finding aligns with our previous observation that Nipped-B localizes along the arms but not at the centromeres of oocyte chromosomes (Gause *et al.* 2008). We have recently reported that newly synthesized cohesin is loaded onto oocyte chromosomes during meiotic prophase and used to form new cohesive linkages (Haseeb *et al.* 2024). Together, our observations support the hypothesis that Brm and Pum activities promote Nipped-B-dependent loading of cohesin onto chromosome arms during prophase rejuvenation in *Drosophila* oocytes.

### Additional interesting observations

Our screen also provided information about gene products required for normal reproductive biology in *Drosophila* females as well as the effect of hairpin insertion site on baseline NDJ in control (no KD) oocytes. Germline knockdown of several proteins severely reduced female fertility or caused complete sterility (Supplementary Table 9). In addition, Supplementary Table 10 lists gene products for which knockdown significantly decreased NDJ compared to the control, suggesting that these proteins negatively impact the fidelity of chromosome segregation when present at normal levels in oocytes. Interestingly, we also observed that control oocytes containing an attP40 hairpin insertion exhibited higher baseline NDJ than those with an attP2 insertion (Supplementary Fig. 5). These data align with other reports that the attP40 insertion site can influence phenotypes in multiple *Drosophila* tissues (Groen *et al.* 2022; van der Graaf *et al.* 2022; Duan *et al.* 2023).

## Conclusions

Our screen, designed to identify proteins required for cohesion rejuvenation in *Drosophila* oocytes, uncovered 29 gene products that have a prophase-specific function required for accurate chromosome segregation. We characterized two prophase-specific positives, Brahma and Pumilio, which have functional links with the cohesin loader Nipped-B. Arm cohesion defects increase significantly when Brahma, Pumilio, or Nipped-B is knocked down during meiotic prophase, indicating that all three proteins are required for the maintenance of arm cohesion in *Drosophila* oocytes. We propose that during prophase in *Drosophila* oocytes, a Brahma-containing chromatin remodeling complex recruits Nipped-B to nucleosome-free regions, facilitating the loading of new cohesin complexes onto chromosome arms. Furthermore, we posit that Pumilio-dependent stabilization of Nipped-B mRNA during meiotic prophase ensures that Nipped-B protein levels are sufficient for rejuvenation. Future analyses will better define the molecular mechanism(s) by which Brahma and Pumilio influence Nipped-B function and determine whether any of the 27 additional prophase-specific positives is required for cohesion rejuvenation. Our validated screening method also could be expanded beyond the 63 targets that we knocked down in this study.

## Supplementary Material

jkae123_Supplementary_Data

## Data Availability

Fly strains are available upon request. The authors affirm that all data necessary for confirming the conclusions of the article are present within the text, figures, and tables. Supplementary Tables 1 and 2 provide details for all the fly stocks used in this study. Supplemental material available at G3 online.
