# Supplementary material for: An RNAi screen to identify proteins required for cohesion rejuvenation during meiotic prophase in Drosophila oocytes
Source: G3 (Bethesda). 2024 Jun 8;14(8):jkae123. doi: 10.1093/g3journal/jkae123 (PMC11304968; doi:10.1093/g3journal/jkae123)
Supplement: jkae123_Supplementary_Data [file jkae123_supplementary_data.zip › Figure_S1_G3-2023-404776.pdf]

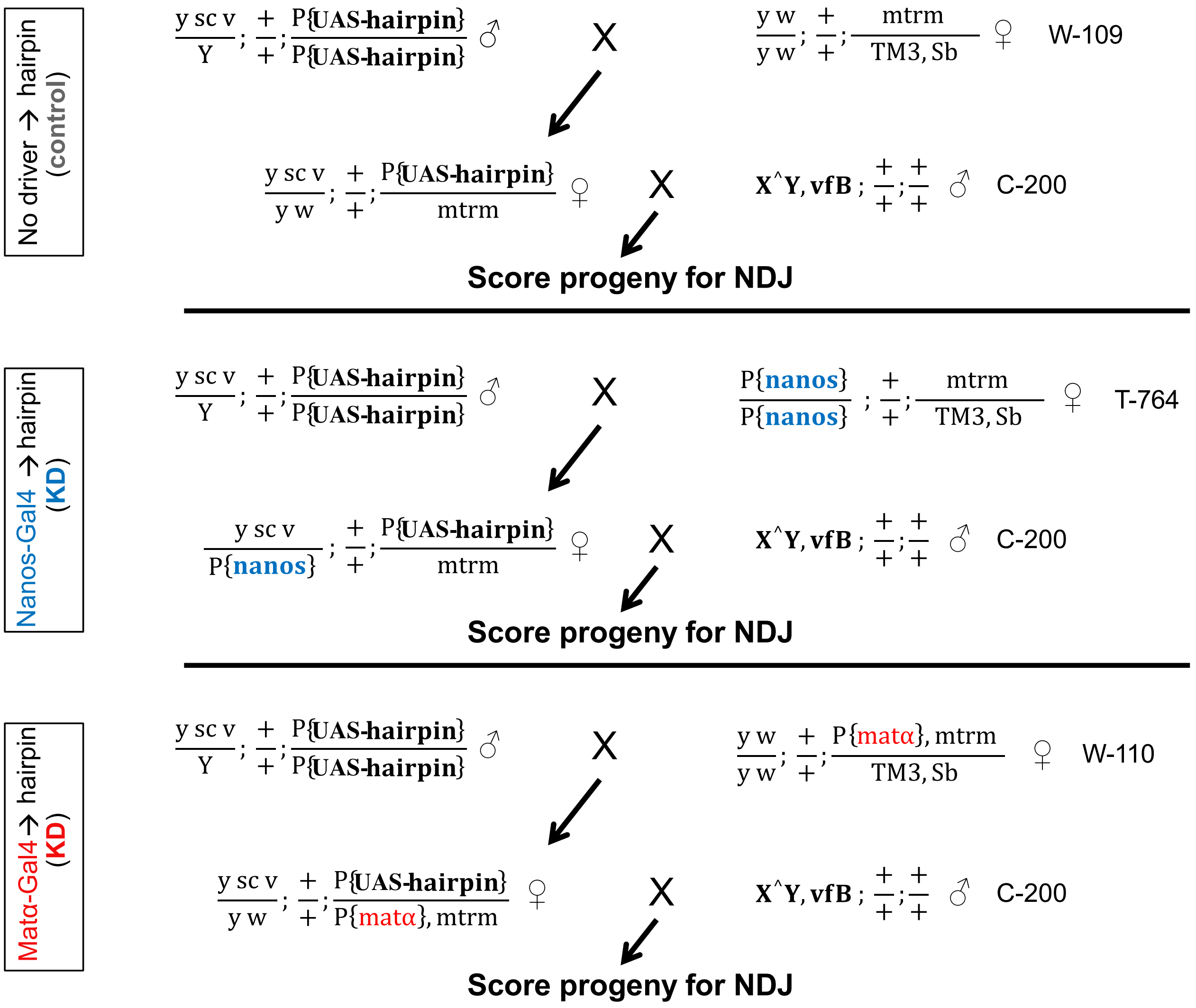

Figure S1. Cross schemes for NDJ screen. Crosses are shown for a hairpin stock with a 3rd chromosome hairpin insertion and an *X* chromosome marked with *y sc v*. 2nd chromosome hairpins were also tested, and the *X* chromosome genotype also varied between hairpin stocks. Attached *X^Y* males were crossed to Control (no driver) or KD oocytes and NDJ scored as shown in Fig 2D
