# Supplementary material for: An RNAi screen to identify proteins required for cohesion rejuvenation during meiotic prophase in Drosophila oocytes
Source: G3 (Bethesda). 2024 Jun 8;14(8):jkae123. doi: 10.1093/g3journal/jkae123 (PMC11304968; doi:10.1093/g3journal/jkae123)
Supplement: jkae123_Supplementary_Data [file jkae123_supplementary_data.zip › Figure_S3_G3-2023-404776.pdf]

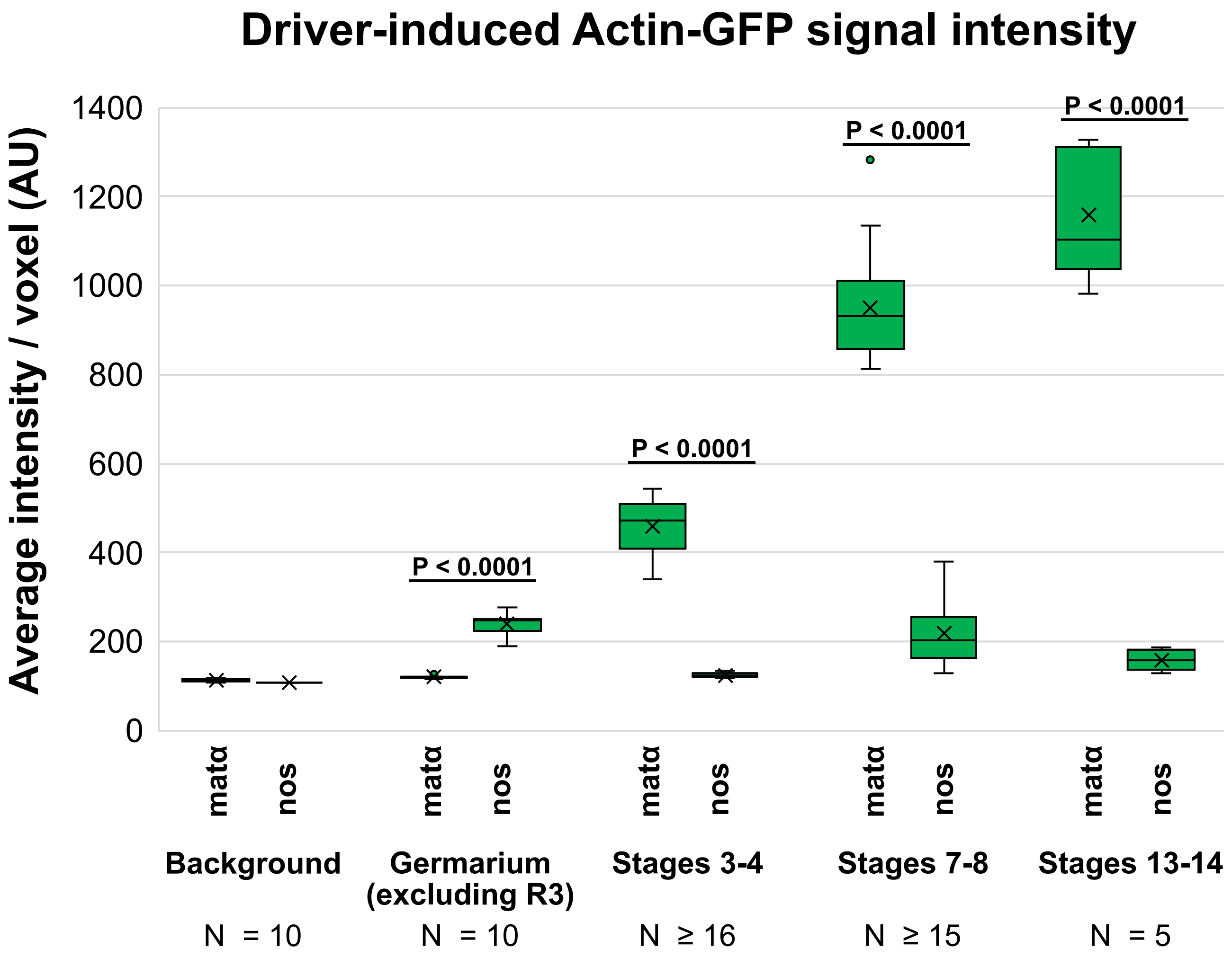

Figure S3. Comparison of matα and nanos driver strengths at different stages. Signal intensity for actin-GFP expression, induced by either the matα or nanos (nos) driver, is shown for the specified stages. Background corresponds to 488 nm signal intensity in areas lacking tissue. Unlike the nanos driver, no matα-induced actin-GFP signal is detectable in the germarium when region 3 is excluded from the measurements (matα-signal intensity is comparable to background). In stages posterior to the germarium, matα-induced actin-GFP signal is detectable, increases during oogenesis, and is significantly higher than nanos-driven expression. The average intensity is indicated by an "X", with median and quartiles depicted by horizontal lines. AU = arbitrary units. P values were calculated using an unpaired *t* test. For each pair of values graphed, the N value is shown below the X axis.
