# Supplementary material for: An RNAi screen to identify proteins required for cohesion rejuvenation during meiotic prophase in Drosophila oocytes
Source: G3 (Bethesda). 2024 Jun 8;14(8):jkae123. doi: 10.1093/g3journal/jkae123 (PMC11304968; doi:10.1093/g3journal/jkae123)
Supplement: jkae123_Supplementary_Data [file jkae123_supplementary_data.zip › Figure_S4_G3-2023-404776.pdf]

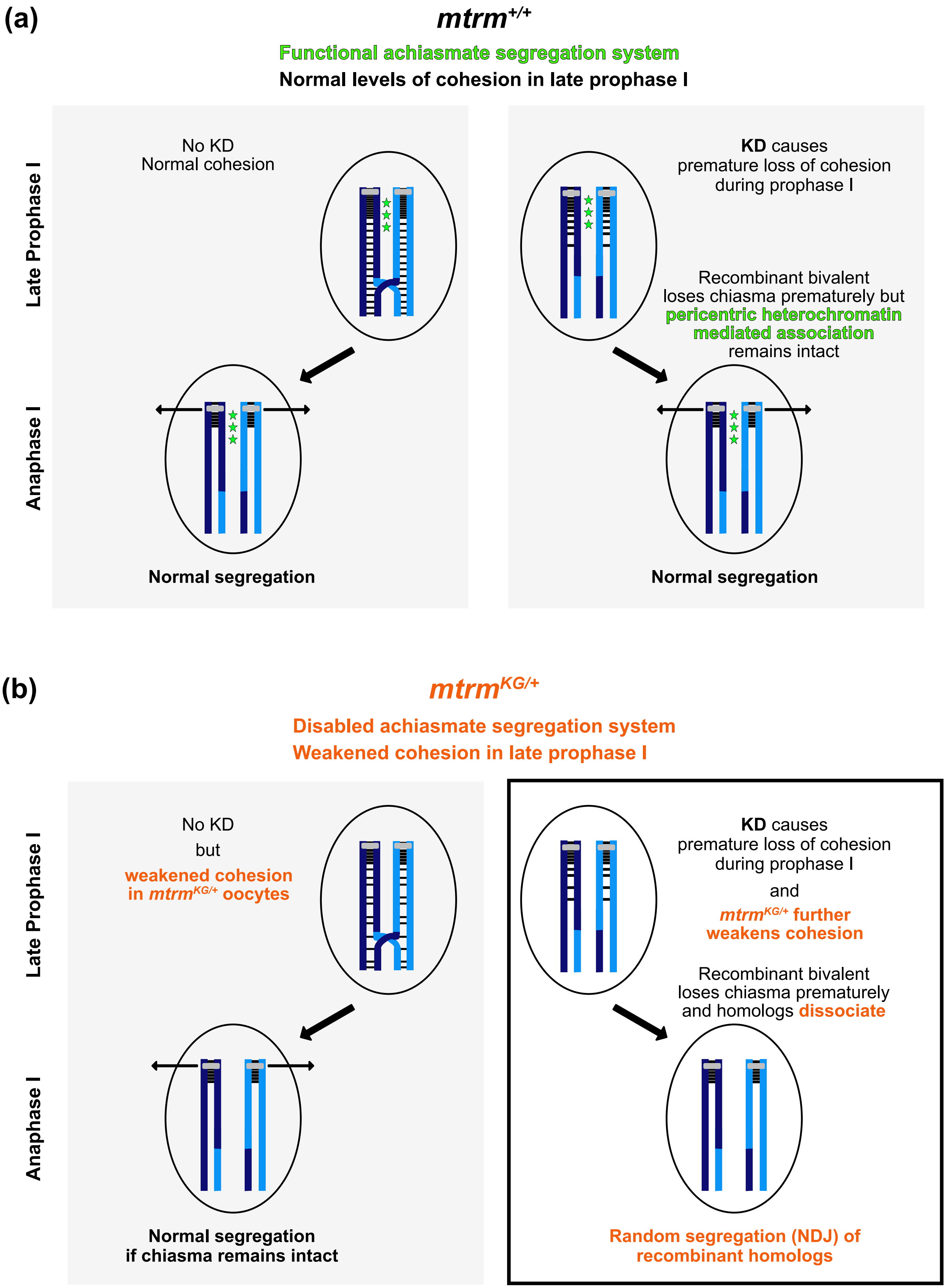
(a)
mtrm+/+
Functional achiasmate segregation system
Normal levels of cohesion in late prophase I
Late Prophase I
Anaphase I
No KD
Normal cohesion
Normal segregation
KD causes
premature loss of cohesion
during prophase I
Recombinant bivalent
loses chiasma prematurely but
pericentric heterochromatin
mediated association
remains intact
Normal segregation
(b)
mtrmKG/+
Disabled achiasmate segregation system
Weakened cohesion in late prophase I
Late Prophase I
Anaphase I
No KD
but
weakened cohesion
in mtrmKG/+ oocytes
Normal segregation
if chiasma remains intact
KD causes
premature loss of cohesion
during prophase I
and
mtrmKG/+ further
weakens cohesion
Recombinant bivalent
loses chiasma prematurely
and homologs dissociate
Random segregation (NDJ) of
recombinant homologs

Figure S4. *mtrm*^{KG} heterozygotes provide a sensitized genetic background to score for NDJ arising from premature loss of cohesion. a) In Drosophila oocytes, pericentric heterochromatin mediated association of homologs (depicted by green stars) keeps achiasmate bivalents physically associated and ensures their proper segregation (left). This same achiasmate system also promotes accurate segregation of recombinant homologs when a chiasma is destabilized due to premature loss of arm cohesion (right). Therefore, knockdown of proteins required for cohesion maintenance may not significantly increase NDJ in *mtrm*$^{+}$ oocytes. b) We performed our screen using oocytes that were heterozygous for the *mtrm*^{KG08051} allele. The achiasmate system is disabled in *mtrm*$^{KG/+}$ oocytes (Harris *et al.* 2003) and cohesion is weakened (but not eliminated) due to increased Polo kinase activity in late prophase (Xiang *et al.* 2007; Bonner *et al.* 2020; Haseeb *et al.* 2023). In the absence of KD, weakened cohesion is still sufficient to promote accurate segregation (left). If KD further disrupts cohesion, premature dissociation of recombinant homologs leads to NDJ (right). Therefore, *mtrm*^{KG} heterozygotes provide an excellent sensitized genetic background to screen for gene products that, upon knockdown, result in premature loss of cohesion.
