# Supplementary material for: An RNAi screen to identify proteins required for cohesion rejuvenation during meiotic prophase in Drosophila oocytes
Source: G3 (Bethesda). 2024 Jun 8;14(8):jkae123. doi: 10.1093/g3journal/jkae123 (PMC11304968; doi:10.1093/g3journal/jkae123)
Supplement: jkae123_Supplementary_Data [file jkae123_supplementary_data.zip › Figure_S5_G3-2023-404776.pdf]

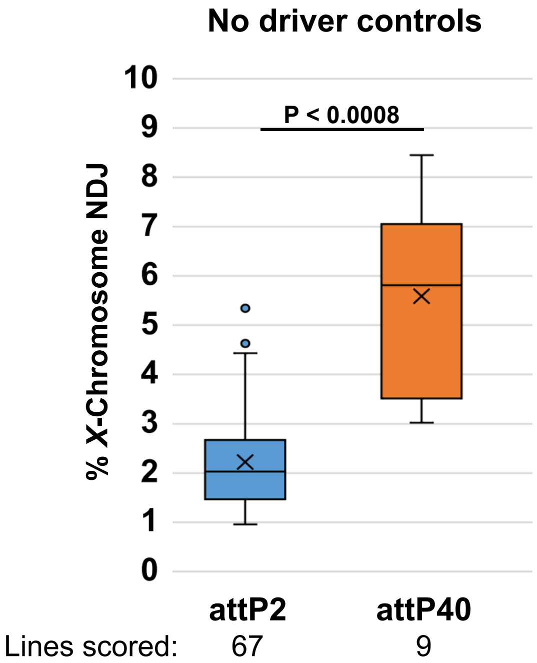

Figure S5. Effect of hairpin insertion site on NDJ in control oocytes. *X*-chromosome NDJ in control oocytes (no knockdown) is significantly higher for oocytes with hairpins inserted at the attP40 site on chromosome *2* than for hairpins at the attP2 site on chromosome *3*. The number of independent insertion lines tested are shown below each insertion site. An unpaired, two-tailed Student's t-test was performed to calculate the P value.
