# Supplementary material for: An RNAi screen to identify proteins required for cohesion rejuvenation during meiotic prophase in Drosophila oocytes
Source: G3 (Bethesda). 2024 Jun 8;14(8):jkae123. doi: 10.1093/g3journal/jkae123 (PMC11304968; doi:10.1093/g3journal/jkae123)
Supplement: jkae123_Supplementary_Data [file jkae123_supplementary_data.zip › Table S9.pdf]

Table S9. Hairpins for which knockdown decreases fertility at least three-fold.

Decreased fertility with matα driver		
**Gene name**	**Control** Fertility	**Matα** Fertility
CG14712	14.3	0.4
CCT3	15.4	1.1
Gwl	17.5	1.7
Smc2	10.7	1.7
Imp	15.9	2.6
Rap1	17.7	3.0
Abl hairpin#1	9.6	1.6
Abl hairpin#2	15.4	4.7
Mps1 hairpin#2	26.5	6.7
**Decreased fertility with nanos driver**		
**Gene name**	**Control** Fertility	**Nanos** Fertility
eIF5	12.2	Sterile
Dhc64C	10.2	Sterile
CCT3	15.4	Sterile
SmB	14.0	Sterile
Smc2	10.7	Sterile
Smc4	13.5	Sterile
CG14712	14.3	0.04
Hip14	15.3	0.04
Gwl	17.5	0.10
Hang	12.4	0.14
AhcyL1	12.2	0.80
Sunn	14.7	4.60
Ord	19.5	5.30

Fertility values indicate number of progeny per female in the NDJ assay.
