# Supplementary material for: An RNAi screen to identify proteins required for cohesion rejuvenation during meiotic prophase in Drosophila oocytes
Source: G3 (Bethesda). 2024 Jun 8;14(8):jkae123. doi: 10.1093/g3journal/jkae123 (PMC11304968; doi:10.1093/g3journal/jkae123)
Supplement: jkae123_Supplementary_Data [file jkae123_supplementary_data.zip › Table_S1_G3-2023-404776.pdf]

Table S1. Fly stocks utilized in primary screen and subsequent assays.

Genotype	Hairpin	Abbreviation	Source	Bickel Stock #
w*; + ; P{w^{+mC}=**matα4**-GAL4-VP16}V37		matα	BL #7063	T-273
y^{1} Df(1)w^{67c23}		y w		A-062
y^{1}; P{y$^{+mDint2}$ w$^{BR.E.BR}$=SUPorP} Exo70^{KG08051} **mtrm^{KG0805}** ry^{506} / TM3,Sb^{1}Ser^{1}			BL #14932	M-755
y w / B^{S} Y ; + ; **mtrm^{KG0805}**/TM3,Sb		mtrm^{KG}	Bickel lab derivative of M-755	W-109
y w / B^{S} Y ; + ; **mtrm^{KG0805}** P{w^{+mC}=**matα4**-GAL4-VP16}V37/TM3,Sb		mtrm^{KG}, matα	Perkins et al. 2016	W-110
P{**nanos**-Gal4-VP16, MVD2 w^{+}} y w/Y; + ; TM3,Ser,Sb/D			Hawley lab	OL-043
P{**nanos**-Gal4-VP16, MVD2 w^{+}} y w/B^{S} Y ; + ; **mtrm^{KG0805}**/TM3,Sb		nanos, mtrm^{KG}	Bickel lab derivative of M-835 & OL-043	T-764
C(1)RM, y^{2}, su(w^{a}) w^{a} / **X^Y,v f B**		X^Y, Bar	BL #700	C-200
w^{1118} ; P{w^{+mC}=**UASp-Act5C.T:GFP}**2; l(3)**/TM6C, Sb^{1} Tb^{1}			BL #7310	B-071
y w; P{w^{+mC}=**UASp-Act5C.T:GFP}**2; +		UASp-Actin-GFP	Bickel Lab derivative of B-071	A-201
**y sc cv v f car**/FM7a/B^{S}Y ; + ; **mtrm^{KG0805}** P{w^{+mC}=**matα4**-GAL4-VP16}V37/TM3,Sb(Ser)		y sc cv v f car; mtrm^{KG}, matα	Perkins et al. 2016	M-834
**y sc cv v f car**/FM7a/B^{S}Y ; + ; **mtrm^{KG0805}**/TM3,Sb(Ser)		y sc cv v f car; mtrm^{KG}	Perkins et al. 2016	M-835
y^{1} sc^{1} v^{1} ; + ; P{y$^{+t7.7}$ v$^{+t1.8}$=TRiP HMS00050=**Brm**^{V20}}attP2	SH00130.N Valium 20	TRiP Brm^{V20}	BL #34520	H-209
y^{1} sc^{1} v^{1} sev^{21} ; + ; P{y$^{+t7.7}$ v$^{+t1.8}$=TRiP HMS01564=**Pum**^{V20}}attP2	SH02112.N Valium 20	TRiP Pum^{V20}	BL #36676	H-211
y^{1} sc^{1} v^{1} ; P{y$^{+t7.7}$ v$^{+t1.8}$= TRiP.GL00574=**Nipped-B**^{V22}}attP40; +	SH002735.N Valium 22	TRiP Nipped-B^{V22}	BL #36614	H-063
y ; + ; P{y$^{+t7.7}$ v$^{+t1.8}$=TRiP HMS00050=**Brm**^{V20}}attP2	SH00130.N Valium 20	y; + ;TRiP Brm^{V20}	Bickel lab derivative of H-209	I-563
y ; + ; P{y$^{+t7.7}$ v$^{+t1.8}$=TRiP HMS01564=**Pum**^{V20}}attP2	SH02112.N Valium 20	y; + ;TRiP Pum^{V20}	Bickel lab derivative of H-211	I-576

BL = Bloomington Drosophila Stock Center
