# Supplementary material for: An RNAi screen to identify proteins required for cohesion rejuvenation during meiotic prophase in Drosophila oocytes
Source: G3 (Bethesda). 2024 Jun 8;14(8):jkae123. doi: 10.1093/g3journal/jkae123 (PMC11304968; doi:10.1093/g3journal/jkae123)
Supplement: jkae123_Supplementary_Data [file jkae123_supplementary_data.zip › Table_S10_G3-2023-404776.pdf]

Table S10. Hairpins for which knockdown reduces NDJ.

**Gene name** (hairpin ID)<br>*Vector, insertion site*	**% *X*-chromosome NDJ**<br>*(Fertility)*			**P value**		
	**Control**	**Nanos KD**	**Matα KD**	**Nanos**<br>&<br>**Control**	**Matα**<br>&<br>**Control**	**Nanos**<br>&<br>**Matα**
**Lsd-2** (SH00412.N)<br>*V20, attP2*	**1.66**<br>*(15.9)*	***0.17**<br>*(15.0)*	**2.47**<br>*(15.0)*	0.0095	0.33	0.00044
**Park** (SH03863.N)<br>*V20, attP2*	**5.34**<br>*(10.9)*	***2.01**<br>*(12.3)*	**8.32**<br>*(12.1)*	0.0077	0.072	<0.0001
**AhcyL1** (SH02584.N2)<br>*V22, attP2*	**3.82**<br>*(12.2)*	**8.57**<br>*(0.80)*	***1.36**<br>*(5.50)*	0.33	0.036	0.14

Fertility values shown in *( )* indicate the number of progeny per female in the NDJ assay. Asterisk indicates a significant difference in NDJ compared to the control (P < 0.05). V20 and V22 are VALIUM 20 and VALIUM 22 vectors respectively.
