# Supplementary material for: An RNAi screen to identify proteins required for cohesion rejuvenation during meiotic prophase in Drosophila oocytes
Source: G3 (Bethesda). 2024 Jun 8;14(8):jkae123. doi: 10.1093/g3journal/jkae123 (PMC11304968; doi:10.1093/g3journal/jkae123)
Supplement: jkae123_Supplementary_Data [file jkae123_supplementary_data.zip › Table_S2_G3-2023-404776.pdf]

Table S2. Original hairpin stocks tested in NDJ screen.

Gene	Hairpin ID	Vector	Insertion site	Source
Abl #1	SH01558.N2	V22	attP2	BL #35327
Abl #2	SH07944.N	V20	attP40	BL #61170
AGBE	SH02397.N2	V22	attP2	BL #42753
AhcyL1	SH02584.N2	V22	attP2	BL #43168
Baz	SH02076.N	V20	attP2	BL #35002
Brm #1	SH00130.N	V20	attP2	BL #34520
Brm #2	SH01379.N2	V22	attP2	BL #35211
BthD	SH03724.N2	V22	attP2	BL #43267
CCT3	SH00729.N	V20	attP2	BL #34969
Cype	SH00317.N	V20	attP2	BL #33878
Dhc64C	SH02710.N	V20	attP2	BL #36698
Diap1	SH00680.N	V20	attP2	BL #33597
EIF5	SH00345.N	V20	attP2	BL #34841
Fmr1 #1	SH00354.N	V20	attP2	BL #34944
Fmr1 #2	SH01274.N2	V22	attP2	BL #35200
Fs(1)k10	SH00087.N	V20	attP2	BL #33630
Grp	SH01903.N	V20	attP2	BL #36685
Gwl	SH00920.N	V20	attP2	BL #34525
Hang	SH02893.N2	V22	attP40	BL #41870
HEM	SH04538.N	V20	attP2	BL #41688
Hip14	SH02106.N	V20	attP2	BL #35012
ifc	SH00391.N	V20	attP2	BL #32514
Imp	SH01803.N	V20	attP2	BL #34977
Iswi	SH00393.N	V20	attP2	BL #32845
Lsd-2	SH00412.N	V20	attP2	BL #32846
Mamo	SH07656.N	V20	attP2	BL #60111
Mbc	SH05347.N	V20	attP2	BL #36658
mCherry	SH02163.N	V20	attP2	BL #35785
Mps1 #1	SH01602.N	V20	attP2	BL #36658

Mps1 #2	shRNA-330259	Walium 20	attP40	VDRC #330259
Ord	SH02738.N2	V22	attP2	BL #36633
pAbp	unavailable	V20	attP2	BL #67979
Park	SH03863.N	V20	attP2	BL #38333
Plc21C	SH00900.N	V20	attP2	BL #33719
Pum #1	SH02803.N	V20	attP40	BL #38241
Pum #2	SH02112.N	V20	attP2	BL #36676
Punch	SH03835.N	V20	attP2	BL #55397
Raf	SH03151.N	V20	attP2	BL #55679
Rap1	SH02250.N	V20	attP2	BL #35047
Ras85D	SH01307.N	V20	attP2	BL #34619
Rbfox1	SH00782.N	V20	attP2	BL #32476
RhoGAP1A	SH00517.N	V20	attP2	BL #33390
RhoGAP92B	SH00519.N	V20	attP2	BL #33391
RYBP	SH00691.N	V20	attP2	BL #33974
Scf	SH01884.N	V20	attP2	BL #34331
Septin 4	SH02657.N2	V22	attP2	BL #41859
SERCA	SH004955.N	V20	attP2	BL #44581
Singed	SH04535.N	V22	attP2	BL #42615
SmB	SH06421.N	V20	attP2	BL #64594
Smc1	SH01950.N2	V22	attP2	BL #36598
Smc2	SH00559.N	V20	attP2	BL #32369
Smc3	SH03166.N	V20	attP40	BL #50899
Smc4	SH00364.N	V20	attP2	BL #32350
Socs44A	SH04771.N	V20	attP2	BL #42830
Sunn	SH020-F10	V20	attP40	BL #52969
Tao	SH01902.N	V20	attP2	BL #34881
Thiolase	SH00737.Nb	V20	attP2	BL #34546
Tos	SH00792.N	V20	attP2	BL #33937
Tre1	SH00895.N	V20	attP2	BL #34956
CG2941	SH02890.N2	V22	attP40	BL #41867

CG4294	SH00243.N	V20	attP2	BL #33357
CG5292 #1	SH00947.N	V20	attP2	BL #32499
CG5292 #2	SH00948.N	V20	attP2	BL #32500
CG6418	SH00920.N	V20	attP2	BL #32441
CG6805 #1	SH00873.N	V20	attP2	BL #32380
CG6805 #2	SH00872.N	V20	attP2	BL #34615
CG7115	SH08133.N	V20	attP2	BL #60015
CG9925	SH01506.Nb	V20	attP2	BL #35811
CG10082 #1	SH00874.N	V20	attP2	BL #32431
CG10082 #2	SH00875.N	V20	attP2	BL #33717
CG10924	SH00155.N	V20	attP2	BL #36915
CG12084	SH01649.N	V20	attP2	BL #34553
CG14712	SH01504.N2	V22	attP2	BL #35622
CG17658	SH01091.N2	V22	attP40	BL #36872
CG18446	SH00207.N	V20	attP2	BL #33735
CG42232	SH07828.N2	V20	attP40	BL #64604

BL = Bloomington Drosophila Stock Center
VDRC = Vienna Drosophila Resource Center
V20 and V22 are TRiP vectors Valium 20 and Valium 22 respectively
