# Supplementary material for: An RNAi screen to identify proteins required for cohesion rejuvenation during meiotic prophase in Drosophila oocytes
Source: G3 (Bethesda). 2024 Jun 8;14(8):jkae123. doi: 10.1093/g3journal/jkae123 (PMC11304968; doi:10.1093/g3journal/jkae123)
Supplement: jkae123_Supplementary_Data [file jkae123_supplementary_data.zip › Table_S3_G3-2023-404776.pdf]

**Table S3.** Gene products for which nanos-induced knockdown causes significantly higher NDJ than the matα driver.

| **Gene name** (hairpin ID)<br>*Vector, insertion site* | **% *X*-chromosome NDJ**<br>*(Fertility)* | | | **P value** | | |
|---|---|---|---|---|---|---|
| | **Control** | **Nanos KD** | **Matα KD** | **Nanos** & **Control** | **Matα** & **Control** | **Nanos** & **Matα** |
| **Smc1** (SH01950.N2)<br>*V22, attP2* | **1.57**<br>*(11.1)* | ***35.24**<br>*(16.4)* | ***6.08**<br>*(16.8)* | <0.0001 | <0.0001 | <0.0001 |
| **Smc3** (SH03166.N)<br>*V20, attP40* | **7.80**<br>*(15.1)* | ***33.69**<br>*(13.5)* | ***18.33**<br>*(13.8)* | <0.0001 | <0.0001 | <0.0001 |
| **Ord** (SH02738.N2)<br>*V22, attP2* | **2.35**<br>*(19.5)* | ***57.19**<br>*(5.30)* | ***5.62**<br>*(17.7)* | <0.0001 | 0.0017 | <0.0001 |
| **Sunn** (SH020-F10)<br>*V20, attP40* | **3.02**<br>*(14.7)* | ***47.62**<br>*(4.60)* | ***9.29**<br>*(16.9)* | <0.0001 | <0.0001 | <0.0001 |
| | | | | | | |
| **Rbfox1** (SH00782.N)<br>*V20, attP2* | **1.69**<br>*(13.2)* | ***34.78**<br>*(13.6)* | ***6.47**<br>*(13.8)* | <0.0001 | <0.0001 | <0.0001 |
| **Mamo** (SH07656.N)<br>*V20, attP2* | **1.73**<br>*(11.5)* | ***15.37**<br>*(14.3)* | ***6.67**<br>*(12.5)* | <0.0001 | <0.0001 | <0.0001 |
| | | | | | | |
| **Negative Control:** | | | | | | |
| **mCherry** (SH02163.N)<br>*V20, attP2* | **2.30**<br>*(15.1)* | **2.18**<br>*(18.2)* | **3.31**<br>*(18.6)* | 0.88 | 0.26 | 0.18 |

*Fertility values* shown in *( )* indicate the number of progeny per female in the NDJ assay. Asterisk indicates a significant difference in NDJ compared to the control ($P < 0.05$). V20 and V22 are VALIUM 20 and VALIUM 22 vectors respectively.
