# Supplementary material for: An RNAi screen to identify proteins required for cohesion rejuvenation during meiotic prophase in Drosophila oocytes
Source: G3 (Bethesda). 2024 Jun 8;14(8):jkae123. doi: 10.1093/g3journal/jkae123 (PMC11304968; doi:10.1093/g3journal/jkae123)
Supplement: jkae123_Supplementary_Data [file jkae123_supplementary_data.zip › Table_S4_G3-2023-404776.pdf]

Table S4. Elevated NDJ is comparable for the two drivers.

**Gene name** (hairpin ID)<br>*Vector, insertion site*	**% *X*-chromosome NDJ**<br>*(Fertility)*			**P value**		
	**Control**	**Nanos KD**	**Matα KD**	**Nanos & Control**	**Matα & Control**	**Nanos & Matα**
**Fmr1** #1 (SH00354.N)<br>*V20, attP2*	**1.56**<br>*(14.3)*	***8.77**<br>*(18.0)*	***10.26**<br>*(9.00)*	<0.0001	<0.0001	0.44
**Fmr1** #2 (SH01274.N2)<br>*V22, attP2*	**3.08**<br>*(12.0)*	***6.45**<br>*(10.1)*	***6.31**<br>*(9.20)*	0.021	0.031	0.935
**BthD** (SH03724.N2)<br>*V22, attP2*	**1.99**<br>*(7.50)*	***6.74**<br>*(10.4)*	***10.18**<br>*(13.1)*	0.0013	<0.0001	0.057
**Ras85D** (SH01307.N)<br>*V20, attP2*	**1.36**<br>*(20.1)*	***9.28**<br>*(20.3)*	***6.96**<br>*(17.7)*	<0.0001	<0.0001	0.10
**Scf** (SH01884.N)<br>*V20, attP2*	**3.96**<br>*(17.3)*	***7.52**<br>*(17.9)*	***8.54**<br>*(17.7)*	0.0039	<0.0004	0.48
**Punch** (SH03835.N)<br>*V20, attP2*	**1.30**<br>*(15.3)*	***4.59**<br>*(16.0)*	***8.18**<br>*(18.8)*	<0.0006	<0.0001	0.0057
**AGBE** (SH02397.N2)<br>*V22, attP2*	**2.40**<br>*(11.3)*	***5.66**<br>*(12.0)*	***6.29**<br>*(10.0)*	0.011	0.0058	0.70
**Raf** (SH03151.N)<br>*V20, attP2*	**2.67**<br>*(11.1)*	***5.44**<br>*(13.9)*	***5.90**<br>*(13.2)*	0.025	0.012	0.74
**Iswi** (SH00393.N)<br>*V20, attP2*	**1.09**<br>*(11.4)*	***5.31**<br>*(10.1)*	***4.58**<br>*(17.6)*	<0.0006	<0.0002	0.59
**CG4294** (SH00243.N)<br>*V20, attP2*	**1.39**<br>*(10.7)*	***3.61**<br>*(12.9)*	***4.78**<br>*(14.3)*	0.03	0.0014	0.33
**CG9925** (SH01506.Nb)<br>*V20, attP2*	**0.96**<br>*(13.0)*	***2.91**<br>*(15.2)*	***3.69**<br>*(17.3)*	0.015	0.0011	0.43

Fertility values shown in *( )* indicate the number of progeny per female in the NDJ assay. Asterisk indicates a significant difference in NDJ compared to the control ($P < 0.05$). V20 and V22 are VALIUM 20 and VALIUM 22 vectors respectively.
