# Supplementary material for: An RNAi screen to identify proteins required for cohesion rejuvenation during meiotic prophase in Drosophila oocytes
Source: G3 (Bethesda). 2024 Jun 8;14(8):jkae123. doi: 10.1093/g3journal/jkae123 (PMC11304968; doi:10.1093/g3journal/jkae123)
Supplement: jkae123_Supplementary_Data [file jkae123_supplementary_data.zip › Table_S5_G3-2023-404776.pdf]

Table S5. Hairpins for which only the nanos driver significantly increases NDJ.

**Gene name** (hairpin ID)<br>*Vector, insertion site*	**% *X*-chromosome NDJ**<br>*(Fertility)*			**P value**		
	**Control**	**Nanos KD**	**Matα KD**	**Nanos & Control**	**Matα & Control**	**Nanos & Matα**
**HEM** (SH04538.N)<br>*V20, attP2*	**2.38**<br>*(10.4)*	***5.48**<br>*(14.7)*	**3.45**<br>*(13.5)*	0.0099	0.32	0.098
**Cype** (SH00317.N)<br>*V20, attP2*	**1.46**<br>*(17.0)*	***4.22**<br>*(20.3)*	**1.46**<br>*(17.0)*	0.0010	0.99	0.0010
**Baz** (SH02076.N)<br>*V20, attP2*	**2.41**<br>*(20.5)*	***4.21**<br>*(18.6)*	**4.10**<br>*(20.3)*	0.048	0.055	0.91

Fertility values shown in *( )* indicate the number of progeny per female in the NDJ assay. Asterisk indicates a significant difference in NDJ compared to the control ($P < 0.05$). V20 and V22 are VALIUM 20 and VALIUM 22 vectors respectively.
