# Supplementary material for: An RNAi screen to identify proteins required for cohesion rejuvenation during meiotic prophase in Drosophila oocytes
Source: G3 (Bethesda). 2024 Jun 8;14(8):jkae123. doi: 10.1093/g3journal/jkae123 (PMC11304968; doi:10.1093/g3journal/jkae123)
Supplement: jkae123_Supplementary_Data [file jkae123_supplementary_data.zip › Table_S6_G3-2023-404776.pdf]

Table S6. NDJ and fertility data for prophase-specific positives.

**Gene name** (hairpin ID)<br>*Vector, insertion site*	**% *X*-chromosome NDJ**<br>*(Fertility)*			**P value**		
	**Control**	**Nanos KD**	**Matα KD**	**Nanos & Control**	**Matα & Control**	**Nanos & Matα**
**CG2941** (SH02890.N2)<br>*V22, attP40*	**8.45**<br>*(11.3)*	**12.02**<br>*(12.3)*	***22.89**<br>*(7.40)*	0.069	<0.0001	<0.0002
**CG42232** (SH07828.N2)<br>*V20, attP40*	**5.81**<br>*(14.6)*	**8.23**<br>*(10.8)*	***20.09**<br>*(11.9)*	0.14	<0.0001	<0.0001
**Abl** #1 (SH01558.N2)<br>*V22, attP2*	**1.55**<br>*(9.60)*	**2.17**<br>*(10.3)*	***17.14**<br>*(1.60)*	0.52	0.0011	0.0018
**Abl** #2 (SH07944.N)<br>*V20, attP40*	**3.97**<br>*(15.4)*	**5.77**<br>*(16.8)*	***15.8**<br>*(4.70)*	0.13	<0.0001	<0.0004
**Hang** (SH02893.N2)<br>*V22, attP40*	**5.67**<br>*(12.4)*	**42.86**<br>*(0.14)*	***16.2**<br>*(10.8)*	0.090	<0.0001	0.23
**CG17658** (SH01091.N2)<br>*V22, attP40*	**6.31**<br>*(12.3)*	**5.57**<br>*(10.9)*	***14.41**<br>*(7.70)*	0.64	<0.0004	<0.0002
**Pum** #1 (SH02803.N)<br>*V20, attP40*	**3.05**<br>*(12.1)*	**4.86**<br>*(10.6)*	***12.27**<br>*(10.3)*	0.168	<0.0001	<0.0001
**Pum** #2 (SH02112.N)<br>*V20, attP2*	**1.32**<br>*(16.9)*	***3.28**<br>*(16.5)*	***6.64**<br>*(20.8)*	0.017	<0.0001	0.0025
**CG5292** #1 (SH00947.N)<br>*V20, attP2*	**3.17**<br>*(12.4)*	**2.87**<br>*(14.6)*	***11.92**<br>*(8.90)*	0.77	<0.0001	<0.0001
**CG5292** #2 (SH00948.N)<br>*V20, attP2*	**1.75**<br>*(12.8)*	**2.15**<br>*(9.20)*	***5.93**<br>*(10.6)*	0.67	0.0011	0.0060
**Grp** (SH01903.N)<br>*V20, attP2*	**4.63**<br>*(11.6)*	**4.26**<br>*(13.2)*	***10.55**<br>*(13.0)*	0.77	<0.0004	<0.0001
**SERCA** (SH04955.N)<br>*V20, attP2*	**4.43**<br>*(11.0)*	**7.08**<br>*(13.3)*	***9.77**<br>*(5.10)*	0.074	0.020	0.26
**pAbp** (no hairpin ID)<br>*V20, attP2*	**2.05**<br>*(13.3)*	**2.51**<br>*(14.8)*	***9.12**<br>*(13.6)*	0.61	<0.0001	<0.0001
**Singed** (SH04535.N)<br>*V22, attP2*	**3.61**<br>*(8.20)*	**4.63**<br>*(10.0)*	***8.37**<br>*(11.2)*	0.49	0.0044	0.026
**Brm** #1 (SH00130.N)<br>*V20, attP2*	**2.39**<br>*(16.6)*	**2.85**<br>*(20.8)*	***7.64**<br>*(19.5)*	0.58	<0.0001	<0.0001
**Brm** #2 (SH01379.N2)<br>*V22, attP2*	**1.68**<br>*(23.6)*	**3.07**<br>*(20.0)*	***3.25**<br>*(22.7)*	0.059	<0.050	0.833

**Mps1** #1 (SH01602.N)<br>*V20, attP2*	**2.28**<br>*(13.0)*	**4.20**<br>*(14.0)*	***6.98**<br>*(13.8)*	0.074	<0.0003	0.043
**Mps1** #2 (VSH330259)<br>*Walium20, attP40*	**6.21**<br>*(26.5)*	**6.58**<br>*(28.4)*	***16.13**<br>*(6.70)*	0.76	<0.0004	<0.0006
**RhoGAP1A** (SH00517.N)<br>*V20, attP2*	**2.13**<br>*(12.8)*	**2.26**<br>*(13.1)*	***6.94**<br>*(19.1)*	0.89	<0.0001	<0.0001
**Plc21C** (SH00900.N)<br>*V20, attP2*	**3.12**<br>*(13.4)*	**3.03**<br>*(13.8)*	***6.74**<br>*(15.8)*	0.93	0.0037	0.0027
**Socs44A** (SH04771.N)<br>*V20, attP2*	**1.99**<br>*(9.90)*	**1.66**<br>*(12.0)*	***6.70**<br>*(13.4)*	0.71	<0.0003	<0.0001
**CG10082** #1 (SH00874.N)<br>*V20, attP2*	**2.58**<br>*(13.4)*	**3.05**<br>*(14.5)*	***6.33**<br>*(17.6)*	0.64	0.0011	0.0048
**CG10082** #2 (SH00875.N)<br>*V20, attP2*	**2.67**<br>*(12.9)*	**1.93**<br>*(15.4)*	**4.69**<br>*(17.2)*	0.412	0.0599	0.0048
**Diap1** (SH00680.N)<br>*V20, attP2*	**2.17**<br>*(12.6)*	**1.32**<br>*(15.1)*	***6.01**<br>*(17.0)*	0.29	<0.0007	<0.0001
**Tao** (SH01902.N)<br>*V20, attP2*	**1.62**<br>*(13.8)*	**2.60**<br>*(15.2)*	***5.75**<br>*(16.0)*	0.24	<0.0002	0.0049
**CG6805** #1 (SH00873.N)<br>*V20, attP2*	**1.42**<br>*(10.5)*	**1.78**<br>*(12.6)*	***5.59**<br>*(13.0)*	0.66	<0.0004	0.0011
**CG6805** #2 (SH00872.N)<br>*V20, attP2*	**3.83**<br>*(14.1)*	**4.32**<br>*(15.3)*	**5.20**<br>*(18.3)*	0.67	0.23	0.45
**CG6418** (SH00920.N)<br>*V20, attP2*	**2.03**<br>*(14.6)*	**3.45**<br>*(14.3)*	***5.41**<br>*(19.3)*	0.14	<0.0008	0.079
**Septin 4** (SH02657.N2)<br>*V22, attP2*	**2.11**<br>*(10.6)*	**1.08**<br>*(11.6)*	***5.36**<br>*(14.5)*	0.22	0.0052	<0.0001
**Tre1** (SH00895.N)<br>*V20, attP2*	**1.16**<br>*(21.5)*	**2.38**<br>*(20.8)*	***5.19**<br>*(22.1)*	0.058	<0.0001	0.0021
**Mbc** (SH05347.N)<br>*V20, attP2*	**1.41**<br>*(10.6)*	**2.00**<br>*(12.4)*	***5.14**<br>*(17.1)*	0.49	0.0003	0.0029
**EIF5** (SH00345.N)<br>*V20, attP2*	**1.83**<br>*(12.2)*	Sterile	***4.91**<br>*(13.4)*	---	0.0058	---
**Dhc64C** (SH02710.N)<br>*V20, attP2*	**0.98**<br>*(10.2)*	Sterile	***4.75**<br>*(5.70)*	---	0.012	---
**CG12084** (SH01649.N)<br>*V20, attP2*	**1.49**<br>*(11.7)*	**2.94**<br>*(14.3)*	***4.72**<br>*(16.5)*	0.11	0.0012	0.10

**RYBP** (SH00691.N)<br>*V20, attP2*	**1.38**<br>*(18.0)*	**2.71**<br>*(17.3)*	***4.64**<br>*(23.2)*	0.077	<0.0001	0.037
**Fs(1)k10** (SH00087.N)<br>*V20, attP2*	**1.47**<br>*(15.2)*	**1.19**<br>*(18.8)*	***4.27**<br>*(20.1)*	0.65	0.0012	0.0002
**Ifc** (SH00391.N)<br>*V20, attP2*	**2.16**<br>*(11.5)*	**3.27**<br>*(13.6)*	***4.24**<br>*(17.3)*	0.28	0.041	0.37
**Hip14** (SH02106.N)<br>*V20, attP2*	**1.30**<br>*(15.3)*	**0.00**<br>*(0.04)*	***4.24**<br>*(16.2)*	--	0.0013	--
**Tos** (SH00792.N)<br>*V20, attP2*	**1.42**<br>*(24.4)*	**2.43**<br>*(21.3)*	***3.88**<br>*(17.1)*	0.12	0.0031	0.11
**CG7115** (SH08133.N)<br>*V20, attP2*	**1.30**<br>*(15.3)*	**1.35**<br>*(14.7)*	***3.18**<br>*(16.3)*	0.94	0.023	0.030

Fertility values shown in *( )* indicate the number of progeny per female in the NDJ assay. Asterisk indicates a significant difference in NDJ compared to the control (P < 0.05). V20 and V22 are VALIUM 20 and VALIUM 22 vectors respectively.
