# Supplementary material for: An RNAi screen to identify proteins required for cohesion rejuvenation during meiotic prophase in Drosophila oocytes
Source: G3 (Bethesda). 2024 Jun 8;14(8):jkae123. doi: 10.1093/g3journal/jkae123 (PMC11304968; doi:10.1093/g3journal/jkae123)
Supplement: jkae123_Supplementary_Data [file jkae123_supplementary_data.zip › Table_S7_G3-2023-404776.pdf]

Table S7. Matα negatives.

**Gene name** (hairpin ID) *Vector, insertion site*	**% *X*-chromosome NDJ** *(Fertility)*			**P value**		
	**Control**	**Nanos KD**	**Matα KD**	**Nanos & Control**	**Matα & Control**	**Nanos & Matα**
**Baz** (SH02076.N) *V20, attP2*	**2.41** *(20.5)*	***4.21** *(18.6)*	**4.10** *(20.3)*	0.048	0.055	0.91
**CG10924** (SH00155.N) *V20, attP2*	**2.24** *(15.4)*	**2.31** *(16.1)*	**2.61** *(18.9)*	0.94	0.66	0.71
**CG18446** (SH00207.N) *V20, attP2*	**1.85** *(10.7)*	**2.11** *(11.7)*	**2.44** *(12.2)*	0.78	0.54	0.73
**Cype** (SH00317.N) *V20, attP2*	**1.46** *(17.0)*	***4.22** *(20.3)*	**1.46** *(17.0)*	0.0010	0.99	0.0010
**HEM** (SH04538.N) *V20, attP2*	**2.38** *(10.4)*	***5.48** *(14.7)*	**3.45** *(13.5)*	0.0099	0.32	0.098
**Lsd-2** (SH00412.N) *V20, attP2*	**1.66** *(15.9)*	***0.17** *(15.0)*	**2.47** *(15.0)*	0.0095	0.33	<0.0005
**Park** (SH03863.N) *V20, attP2*	**5.34** *(10.9)*	***2.01** *(12.3)*	**8.32** *(12.1)*	0.0077	0.072	<0.0001
**RhoGAP92B** (SH00519.N) *V20, attP2*	**4.35** *(11.9)*	**2.86** *(12.9)*	**3.46** *(12.8)*	0.24	0.50	0.58
**SmB** (SH06421.N) *V20, attP2*	**2.81** *(14.0)*	Sterile	**2.62** *(12.2)*	--	0.85	--
**Smc4** (SH00364.N) *V20, attP2*	**2.75** *(13.5)*	Sterile	**3.44** *(6.40)*	--	0.61	--
**Thiolase** (SH00737.Nb) *V20, attP2*	**1.32** *(13.2)*	**0.64** *(15.6)*	**1.87** *(15.9)*	0.25	0.45	0.048

Fertility values shown in *( )* indicate the number of progeny per female in the NDJ assay. Asterisk indicates a significant difference in NDJ compared to the control ($P < 0.05$). V20 and V22 are VALIUM 20 and VALIUM 22 vectors respectively.
