# Supplementary material for: An RNAi screen to identify proteins required for cohesion rejuvenation during meiotic prophase in Drosophila oocytes
Source: G3 (Bethesda). 2024 Jun 8;14(8):jkae123. doi: 10.1093/g3journal/jkae123 (PMC11304968; doi:10.1093/g3journal/jkae123)
Supplement: jkae123_Supplementary_Data [file jkae123_supplementary_data.zip › Table_S8_G3-2023-404776.pdf]

Table S8. Best described cellular function for each prophase-specific positive.

Gene name	Cellular function
CG10082	Predicted kinase activity
Abl	Kinase activity
Grp	Kinase activity
Mps1	Kinase activity
Tao	Kinase activity
CG6805	Predicted phosphatase activity
CG7115	Predicted phosphatase activity
pAbp	mRNA binding
Pum	mRNA binding
Brm	Chromatin remodeling
CG12084	Predicted ubiquitination
Diap1	Ubiquitination
RYBP	Ubiquitination
Socs44A	Ubiquitination
Fs(1)k10	Gametogenesis
Ifc	Gametogenesis
Mbc	GTPase activity
RhoGAP1A	GTPase activity
Septin 4	GTPase activity
Plc21C	Calcium ion homeostasis
SERCA	Calcium ion homeostasis
CG5292	Miscellaneous - Predicted tRNA deaminase activity
CG6418	Miscellaneous - Predicted RNA helicase
Singed	Miscellaneous - Actin binding
Tre1	Miscellaneous - G Protein coupled receptor activity
Tos	Miscellaneous - DNA exonuclease
CG2941	Unknown
CG42232	Unknown
CG17658	Unknown
